# The Effect of Nanoparticle Shape, Orientation, and Heterogeneity on the Optical Birefringence of Polymer Nanocomposites

**DOI:** 10.1021/acs.jpcc.5c07736

**Published:** 2026-02-16

**Authors:** Chen Li, Sathya Edamadaka, Ethan Glor, Melissa J. Vettelson, Nathaniel S Watkins, Jacob Faber-Rico, Russell J. Composto, Robert C. Ferrier, Zahra Fakhraai

**Affiliations:** † Department of Chemistry, 6572 University of Pennsylvania, Philadelphia, Pennsylvania 19104, United States; ‡ Department of Materials Science and Engineering, 6572 University of Pennsylvania, Philadelphia, Pennsylvania 19104, United States; § Department of Chemical Engineering and Materials Science, 3078 Michigan State University, East Lansing, Michigan 48824, United States

## Abstract

Embedding plasmonic nanoparticles (NPs) into polymer nanocomposites (PNCs) is a facile method for integrating them into functional devices, whose properties are tunable through varying NP size, shape, and loading. Using anisotropic NPs adds an additional degree of tunability to their orientation order in the PNC, as properties such as conductivity and charge transport can be enhanced in specific directions. In thin films, the film thickness and block copolymer self-assembly can affect the degree of NP orientation, which can be used as a method of control over these properties. However, large-scale control of orientation order in randomly distributed NPs, with both anisotropic NP shapes and heterogeneous shape distributions, remains a challenge. This is partly due to the lack of cost-effective, ensemble-level characterization methods that can independently determine the orientation order and degree of aggregation of anisotropic NPs. Here, we model the complex index of refraction of PNCs with plasmonic NP inclusions in the optical frequency domain by using an effective medium approximation. We quantitatively relate the simulated optical birefringence of the medium to the orientation order parameter of plasmonic nanorods and nanodisks in a robust manner insensitive to heterogeneity in simulated NP size and shape. Experimentally, we measure this orientation order parameter through the birefringent index of refraction using variable-angle spectroscopic ellipsometry (VASE). We demonstrate that we can independently determine the orientation order and degree of aggregation for various PNCs with gold nanorods and nanosphere inclusions. This facile technique provides a powerful method to broadly measure the average orientation order of anisotropic particles in PNCs, which can be correlated to their functional properties.

## Graphical abstract

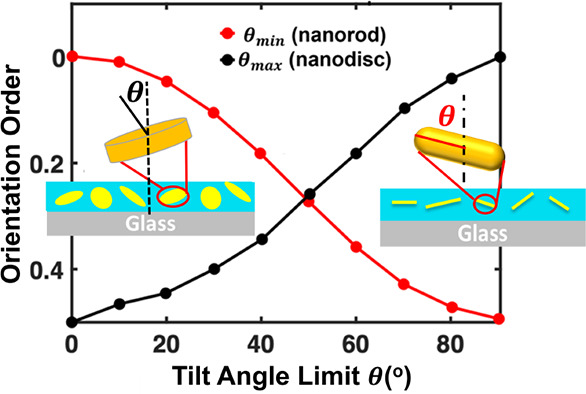

## Introduction

Noble metal nanoparticles (NPs) that self-assemble in polymer nanocomposites (PNCs) are excellent candidates for designing novel coatings. This is due to their low cost, high processability, and facile bottom-up fabrication. Furthermore, unlike precisely controlled ordered packings, randomly self-assembled plasmonic materials with heterogeneous size distributions have material properties that are uniform at large-scale and are thus insensitive to defects.^1–4^ The addition of metal NPs has been shown to produce PNCs with improved mechanical,^5–7^ electrical,^8,9^ and optical^10^ properties, which rival their top-down, precisely designed counterparts whose fabrication relies on costly lithography-etch processes. When anisotropic nanoparticles are utilized, material properties can be tuned by controlling the orientation of the NPs.^11,12^ Control of orientation order can be achieved through film thickness, which can impose in-plane orientation of NPs;^13^ block copolymer self-assembly, which can direct NPs into preferred domains and control both their distance and orientation;^14–16^ or external stimuli, such as light and mechanical shear.^17^ In these systems, both the average orientation order of the NPs^18–20^ and their spatial distribution (i.e., NP proximity and degree of aggregation)^6^ can affect light and heat propagation in various directions. Thus, to predict the final material properties, it is important to readily evaluate and control the orientation order and aggregation state of NP inclusions.

Scanning and transmission electron microscopy (SEM and TEM) have been broadly used to probe the orientation of NPs in polymer nanocomposites.^21–24^ However, the use of TEM is limited to small samples with naturally high variance in measured properties. Obtaining averaged properties from larger-scale experiments can be time-consuming. In two-dimensional SEM projection images, it is also difficult to distinguish between the NP size variations and the distribution of out-of-plane orientation angles. Furthermore, the electron beam may interact with samples in an unintended way, obfuscating the true, as-prepared state of the PNCs. TEM and similar imaging methods are also inherently ex situ characterization techniques and are not able to provide information on the evolution of properties when the material responds to external stimuli such as temperature, light, or solvent exposure.

Single particle microscopy, UV–visible (UV–vis) extinction measurements,^25,26^ infrared spectroscopy, and small-angle X-ray scattering^19^ can also be used to evaluate the orientation order of NPs, both as ex situ and in situ characterization techniques. For example, the effect of doping liquid crystal compounds with nanoparticles, the dispersion of NPs, and their optical properties in these systems have been extensively measured through these methods.^27–29^ In general, single particle measurement techniques, while providing a wealth of detailed information, lack ensemble-level information on heterogeneity in shape, size, aggregation state, and orientation order. X-ray diffraction techniques require large-scale facilities for accurate measurements and have the potential to damage samples. In fact, a low-cost UV–vis birefringence spectrometry approach has been shown to obtain more accurate measurements of high-orientation order parameter specimens than two-dimensional X-ray diffraction spectroscopy,^30^ but this method requires bulk-like samples for contrast and does not work as well in ultrathin films or at low NP loading.

In situ spectroscopic ellipsometry (SE) can overcome many of these challenges. Compared with more commonly used UV–vis reflection or transmission spectroscopy, the inclusion of phase information in the measured reflection coefficients allows the technique to work well at low light intensity. As such, SE can measure the complex index of refraction and thus dielectric constant of the medium in small quantities of the composite material, such as nanometer-sized films.^13,31^ The imaginary part of the index of refraction in SE measurements is proportional to the bulk extinction coefficient of the material, enabling direct measurement of the localized surface plasmon resonance (LSPR) of plasmonic NP inclusions.^13,31^ The LSPR is sensitive to the size,^32,33^ shape,^34–38^ dielectric environment,^31^ and optical coupling of NPs within an ensemble.^39–41^ Properties such as the occupation density,^42^ and the effective dielectric constant^43^ of isotropic metallic NPs coated on a substrate can be readily obtained from the measured complex index of refraction.^31^

We have previously demonstrated that using variable angle SE (VASE), one can also measure the orientation order parameter of gold nanorods (AuNRs) in PNC thin films through the film’s optical birefringence.^13^ Using simple calculations of the dipole moment of a single gold nanorod (AuNR), along with an effective-medium approximation, the measured birefringence of the PNC was then related to the ensemble-averaged orientation order parameter of the embedded AuNRs.^13,44,45^ Furthermore, these experiments demonstrated that SE can be employed in situ to monitor the evolution of the AuNR shape and orientation order parameter upon heating the polymer matrix to above its glass transition temperature *T*_g_.^13^ Reshaping and aggregation result in blue- or red-shifting of the AuNRs extinction spectrum and thus the imaginary part of the index of refraction, while reorientation results in independent changes in the degree of birefringence.

More recently, we have demonstrated the feasibility of this approach for in situ monitoring of block copolymer self-assembly through changes in film thickness and optical birefringence upon solvent uptake and self-assembly,^46^ and imbibition of polymers into porous gold films^47^ as well as self-assembled films of transparent NPs such as silica.^48,49^ This method can also be extended to study the kinetic absorption–desorption of gases on plasmonic NPs^31^ and the thermal stability and optical properties of a host of other organic–inorganic hybrid materials.^22,50–53^ While these measurements demonstrate the power of spectroscopic ellipsometry as a technique to study properties of hybrid materials, many simulations and theoretical models assume perfectly shaped and homogeneous particle properties.^9,13,54^ As such, the robustness of this approach in systems with large heterogeneity of NP sizes and shapes, which is typically the case in experimental systems, is not fully clear.

Here, through modeling and experiments, we demonstrate that even when size heterogeneity is included, the measurements of orientational anisotropy remain robust and reasonably insensitive to a broad range of heterogeneity in the NP length and diameter. We model the effective-medium birefringent indices of refraction of PNCs with the collective optical response from a random, low-density distribution of NPs. Calculations are performed with AuNRs having random distributions of out-of-plane orientations and a Gaussian distribution of NP lengths and diameters. We show that while increasing heterogeneity results in the broadening of the LSPR absorption peak, measured through the imaginary part of the index of refraction, the measured birefringence remains sensitive to the average orientation order of the NPs. We estimate the margin of experimental error when using this approach by assuming experimentally feasible size distributions in simulations. We extend these calculations to nanodiscs, where the axis of symmetry is in the direction normal to the disc plane, along the short axis of the particle, making predictions for future experiments on these systems. In experiments, we demonstrate the feasibility of this approach in systems where the extent of the orientation order parameter can be otherwise estimated. We show that moderate side-by-side or end-to-end aggregation of AuNRs, measured through changes in LSPR resonance, does not affect the measured values of birefringence in thin PNC films, further demonstrating the robustness of this approach. Combining experiments and simulations, we relate the experimentally calculated orientation order parameters to calculated distributions of NP orientation in a robust and predictable model.

## Methods

### Experimental Details

Thiol-terminated polystyrene (PS) with 11.5 and 20 kg/mol molecular weights was purchased from Polymer Source, Inc. and was used without further purification. Poly­(methyl methacrylate) (PMMA) with 1.1 and 77 kg/mol molecular weights and PS with 12 and 20 kg/mol molecular weights for the PNC matrix were purchased from Sigma-Aldrich. Colloidal spheres with 10 nm diameter were purchased from Ted Pella and used as is.

AuNRs were synthesized using an aqueous seed-mediated growth method following previously reported procedures.^25,55–57^ Thiolated PS brushes were grafted to the surface of the AuNRs as described in previous work.^26^ To make PNC solutions, grafted AuNRs were added to solutions of each polymer in toluene and spun-cast onto cleaned Si wafer, clear glass, and frosted glass substrates for SEM, UV–vis, and VASE measurements, respectively, as shown for example in Figure 1. The samples were allowed to dry overnight prior to any imaging or spectroscopy. More details of AuNR synthesis and characterization can be found in the SI. UV–visible spectra were also measured in colloidal solutions, to determine the LSPR resonances of the AuNRs. The two LSPR peaks were measured at 520 nm for the transverse and 741 nm for the longitudinal LSPR, respectively (Figure 1c).

**Figure 1:**
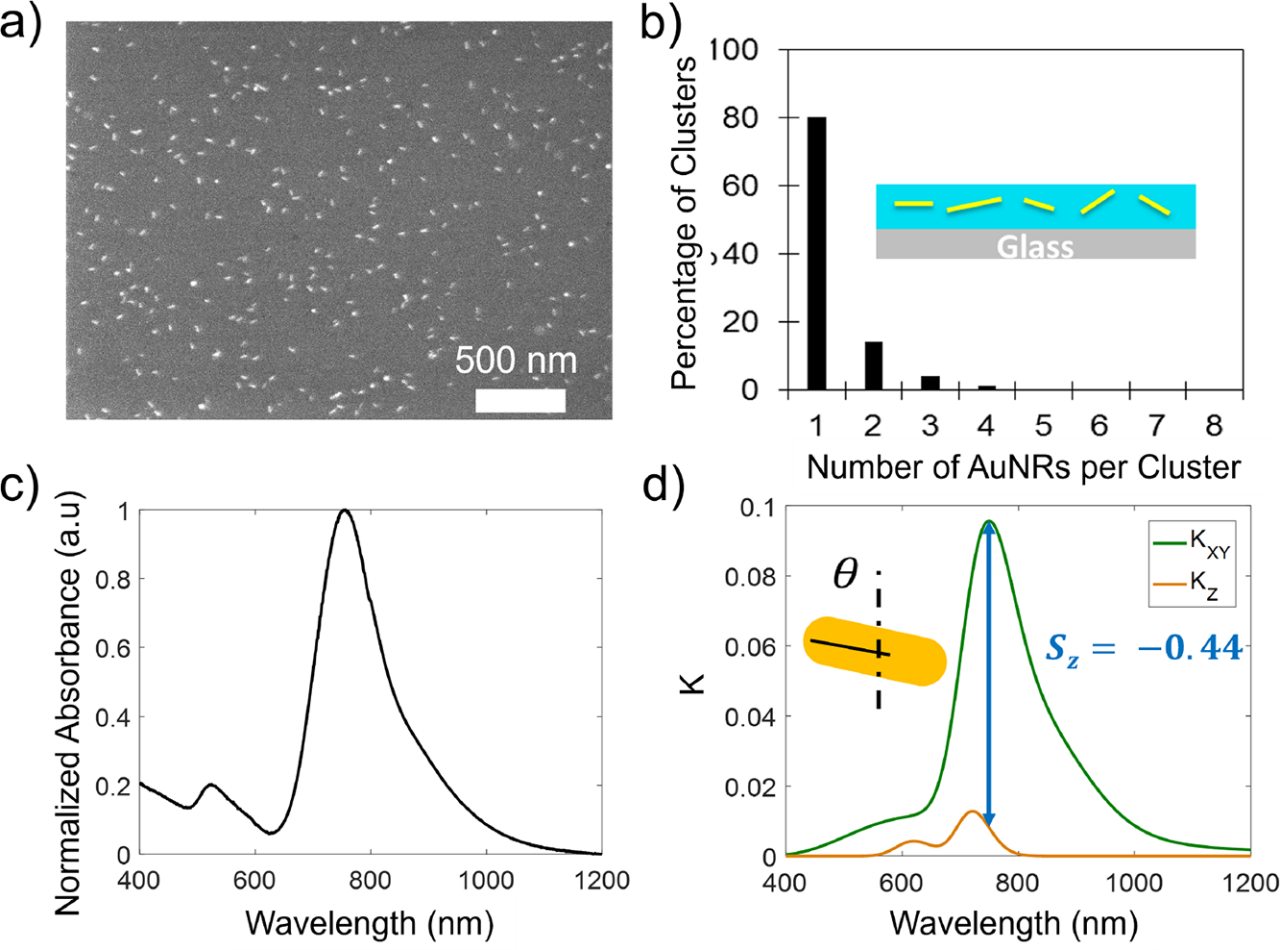
(a) SEM image of a PNC thin film with 4% loading of AuNRs with 20 kg/mol PS brush in a 1.5 kg/mol PS matrix. The average rod length and diameter in this image are measured to be *L* = 38 ± 4.5 and *D* = 11 ± 2.5 nm, respectively. (b) Aggregation statistics of the AuNRs within the composite obtained based on SEM images. The inset shows a schematic side view of the AuNR orientational distribution. (c) UV–visible measurement of the AuNRs in solution with 20 kg/mol PS brush in colloidal state. The longitudinal LSPR peak is measured to be at 741 nm. (d) Extinction coefficient (i.e., the imaginary part of the index of refraction *(K)*) vs wavelength obtained from VASE. The LSPR matches the UV–vis data in (c). The difference between the in-plane (*K*_*xy*_, green) and out-of-plane (*K*_*z*_, orange) indices of refraction is used to evaluate the orientation order parameter *S*_*z*_(SE) = −0.44 ± 0.02, meaning that nearly all of the AuNRs are laying in the plane of the film. The corresponding estimated average tilt angle based on *S*_*z*_(SE) is θ_min_ = (68 ± 5)°. The film thickness is measured to be *h* = 12.1 ± 0.5 nm.

### Scanning Electron Microscopy

Thin film PNCs were analyzed by scanning electron microscopy (SEM) on a JEOL7500F instrument in low-angle backscatter (LABE) mode or secondary emission (SEI) modes. Figure 1a shows an example of SEM measurements performed on a thin PNC film. This data indicates that the AuNRs are generally well dispersed with random orientations in the plane of the film. However, the out-of-plane tilt angle distribution is arbitrary and unknown, as schematically plotted in the insets of Figure 1b. To obtain the AuNR size distributions, measurements were taken both in the film state, as shown in Figure 1a, and on a solid Si wafer substrate. The measured AuNR dimensions were nearly identical in both cases, with the values in thin films being *L* = 38 ± 4.5 and *D* = 11 ± 2.5 nm, whereas on the Si substrate being *L* = 39 ± 4.1 and *D* = 11 ± 1.3 nm.

The aggregation statistics were also obtained by analyzing SEM images. Generally, for PS brushes in the PS matrix, little aggregation was observed. For example, in Figure 1b, it can be seen that though minor aggregates still exist, the nanorods are well dispersed within the PS matrix. The degree of aggregation depends on both the matrix molecular weight and loading of AuNRs, as shown for example in Figure S12 of Supporting Information where aggregation tendency increases with increasing matrix molecular weight. However, even in these systems, the polymer brush enforces some distance between AuNRs, resulting in plasmonic properties that can be well-fitted to dipolar oscillator models as detailed in the next section.

### Spectroscopic Ellipsometry Experiments

Variable-angle spectroscopic ellipsometry (VASE) was performed using a Woollam M200 ellipsometer equipped with focusing optics. Spectroscopic angles Ψ and Δ, which relate to the complex amplitude reflective coefficients of p- and s-polarized light as

$$\frac{r_{p}}{r_{s}}=\tan(\Psi )e^{i\Delta }$$ 1

were measured in the spectral range of 450–1600 nm at five incident angles ranging between 55° and 70° at 5° degree increments. The thickness of each PNC composite was measured using a film cast on Si substrates, and the data were fitted to a transparent polymer model. Due to the low loading of AuNRs and the high reflection coefficient of silicon, AuNRs are hard to detect in these samples. As such, this simplifies the fitting procedure by assuming transparent properties for the polymer matrix, allowing accurate measurements of the film thickness and indices of refraction for PS (*n* = 1.59) and PMMA (*n* = 1.49).

Samples with the same conditions were then made on frosted glass substrates, with the film thickness and index of refraction initially chosen based on the measurements on the silicon substrate. The data in the transparent spectral region (wavelength larger than 1000 nm) were again fitted using these initial values to confirm the accuracy of the film thickness. Given the low reflection coefficient of the glass substrate and the polymer matrix, the majority of the signal in the spectral region of the LSPR originates from the scattering of AuNRs, assumed here to be point dipole inclusions. The frosted glass was used to avoid additional reflections from the backside of the substrate. The details of SE fitting to multiple oscillator models were kept similar to our previous reports^13^ and SI.

Figure S10 shows an example of the raw ellipsometry data and isotropic fitting results. The complex index of refraction, *n*(*k*) = *N*(*k*) + *iK*(*k*), where *k* is the wavevector of the incident light (inversely related to the wavelength) was then calculated based on the measured complex dielectric constant (ϵ­(*k*) = ϵ′(*k*) + ϵ″(*k*)) as $n=\sqrt{\epsilon }$ for nonmagnetic materials. The imaginary part of the index of refraction (*K*) is also the extinction coefficient of the material.^13,58^ As seen in the comparison between Figure 1c,d, the spectral shape and the resonance wavelength of the calculated extinction coefficient from SE fitting agree well with the corresponding UV–visible data obtained from colloidal solutions of grafted AuNRs. The data was then fit to a birefringent model, using the LSPR resonance amplitude as the only fitting parameter, keeping the resonance location and breadth constant as detailed in SI. Figure 1d shows the calculated in-plane (*K*_*xy*_) and out-of-plane (*K*_*z*_) extinction coefficients for a ∼12 nm PNC film, where the majority of AuNRs are expected to be in-plane, resulting in a much smaller amplitude of extinction in the out-of-plane direction (*K*_*z*_). Figure S11 shows additional examples of the calculated real and imaginary parts of the anisotropic indices of refraction for two example PNCs, with varying degrees of optical birefringence, induced by variations of the matrix molecular weight.

### Simulation Details

The optical properties of single gold nanorods (AuNR) and nanodiscs (AuND) were modeled by finite-difference time-domain (FDTD) simulations using the commercial Lumerical FDTD Solutions package (version 8.11). The details of the simulation box and setup are described in the online Supporting Information (SI), as well as our previous publications.^1,13,36,59^ Figure S1 of the Supporting Information shows an example of the simulation setup used for these calculations, along with the calculated extinction coefficients for an AuNR with a diameter of *D* = 12 nm and length of *L* = 34 nm, for illumination in the longitudinal (electric field polarized along the length of AuNR), and transverse (polarized normal to the AuNR length) orientations. The simulated data was then used to calculate the corresponding dipole moments in the longitudinal and transverse directions, as shown in Figure 2b,c, respectively. Details of the dipole moment calculations can be found in the SI. Similar calculations for nanodiscs were performed on a modeled gold cylinder with a diameter of *D* = 30 nm and thickness of *L* = 10 nm (Figure S2).

**Figure 2:**
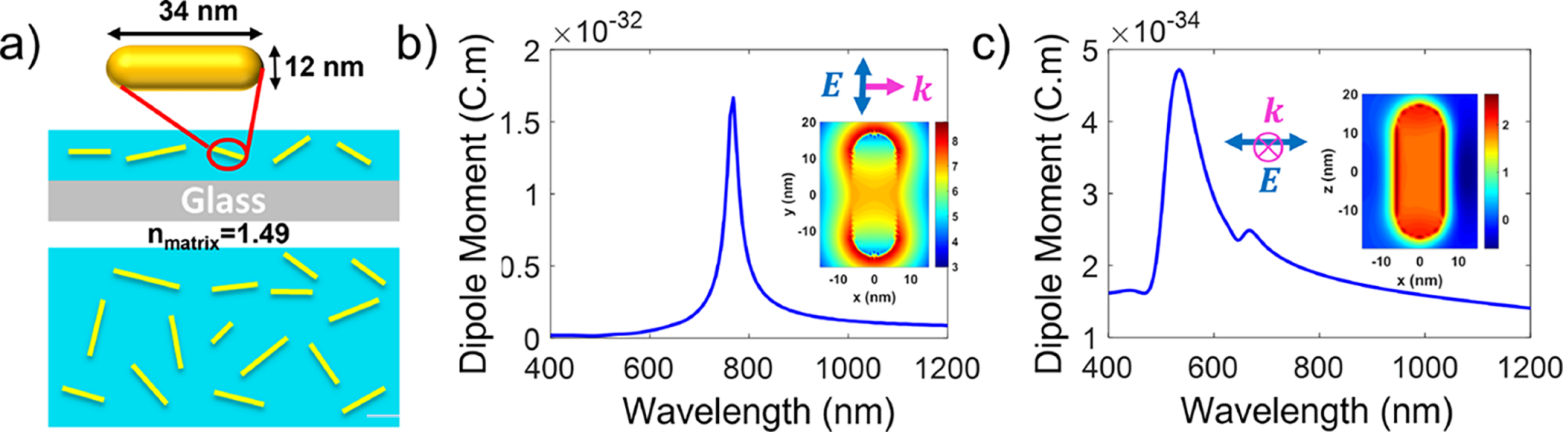
Structure and optical properties of AuNR/polymer composites. (a) A schematic plot of the side- and top-view of heterogeneously sized AuNRs, randomly dispersed in a polymer matrix. The average AuNR length and diameters are *L* = 34 nm and *D* = 12 nm, respectively. (b) The calculated longitudinal and (c) transverse dipole moments of a single AuNR, based on FDTD simulations of the far-field extinction spectra with polarization along the long axis (longitudinal, b) and short axis (transverse, c) of the modeled AuNR, respectively. The inset plots show the near-field distribution of the electric field intensity around the rod at the corresponding resonance wavelengths in a cross-sectional plane through the rod center. The color bars of the insets are in logarithmic scale, normalized to the intensity of the incident electric field (*note: the cartoon is not drawn to scale to demonstrate that rods are not perfectly parallel to the substrate).

We calculated the average dipole moments, extinction spectra, and effective indices of refraction of an ensemble of AuNRs with heterogeneous size dispersions, as schematically shown in Figure 2a. FDTD simulations and dipole moment calculations were performed on AuNRs of various sizes (33 nm < *L* < 42 nm) and diameters (12 nm < *D* < 14 nm) as shown in Figure S3. The LSPR resonances are listed in Table S1. These sizes were chosen based on the experimental size dispersions as detailed in the previous section and previous publications.^13,26^ It was assumed that since the NPs were well dispersed within the polymer matrix, the plasmon-enhanced near-field coupling between adjacent rods was negligible (the validity of this assumption will be discussed in the Results and Discussions section). As such, the spectra were added linearly, with a Gaussian weight, based on the aspect ratio of AuNRs around the average-sized AuNR (*L* = 34 and *D* = 12 nm). The aspect ratio was used as a figure of merit because the LSPR peak changes similarly upon increasing *L* or decreasing *D* as shown in Figure S3. More details of these calculations can be found in the SI. Figure S4 shows how the calculated longitudinal and transverse dipole moments vary as the size dispersion is increased by increasing the width of the Gaussian probability distribution function. The maximum distribution width (Figure S7) was chosen based on experimentally measured size dispersions and extinction spectra.

### Calculations of the Effective Index of Refraction

#### Effective-Medium Approximation

Based on the simulations above, the birefringent index of refraction of a medium containing AuNRs, or nanodiscs (AuNDs), can be calculated to compare with the experimental results from spectroscopic ellipsometry.^13,31^ Given that the NPs used in this study are much smaller than the optical wavelength of light, it can be assumed that the optical response of a PNC film containing well-dispersed NPs is analogous to that of an ensemble of individual point dipoles with weak interactions. The insets in Figure 2b,c show the field maps of the excited AuNRs exhibit predominantly dipolar features, with high electric field intensities at the tips and sides of the rod, respectively, lending credence to the validity of these assumptions. This also implies that the nanoparticles are assumed to be well-dispersed, with a low enough loading density that their near-field interactions are negligible and thus they do not generate significant plasmonic hot spots. This is evident when comparing the solution-based UV–vis spectra in dilute conditions (Figure 1c) and the calculated VASE extinction coefficients (Figure 1d, for example), which have similar spectral shapes and breadths. This assumption works well if the average distance of NPs is larger than their length, which is most often the case even in moderate aggregation. For example, data in Figures S12 and S13 of the SI are fitted based on multiple oscillator models, despite the side-by-side (Figure S12) and end-to-end (Figure S13) aggregation, demonstrating the feasibility of this approach and validating our assumption.

In a thin film, where AuNRs are confined by the composite film’s thickness, predominantly in-plane orientation can be assumed for the nanorods, and thus the longitudinal mode should more easily be excited by the in-plane incident polarization of light. As seen in the comparison between Figure 2b,c, at resonance, the dipole moment of the longitudinal LSPR is about 2 orders of magnitude stronger than that of the transverse LSPR. This asymmetric distribution gives rise to measurable uniaxial optical anisotropy (birefringence) as observed in SE experiments (Figure 1d for example).^13^

The effective refractive index for an ensemble of randomly distributed dipoles all with the same orientation and with identical polarizability (same size), can be described by the Clausius–Mossotti relationship:^60,61^

$$n_{\mathrm{eff}}=\sqrt{\frac{1+\frac{2\rho }{3\epsilon_{0}}\alpha }{1-\frac{\rho }{3\epsilon_{0}}\alpha }}$$ 2

where ρ is the number density of the dipoles in the medium, ϵ_0_ is the free-space permittivity, and α = |**p**|/|**E**_0_| is the polarizability of the dipole (NPs here) under the external field **E**_0_.

#### Calculation of Optical Anisotropy in a Uniform Medium

The optical response of anisotropic particles depends on their relative orientation with respect to the incident polarization. For the AuNRs and AuNDs investigated in this work, the polarizability of induced dipoles under light excitation can be described by a tensor with two independent values of longitudinal (α_l_) and transverse (α_t_) polarizabilities. The polarizability tensor can be written as

$$\left(\begin{array}{ccc} \alpha_{t} & 0 & 0\\ 0 & \alpha_{t} & 0\\ 0 & 0 & \alpha_{l} \end{array}\right)$$ 3

The effective permittivity tensor can be obtained by applying the Clausius–Mossotti relation to the above polarizability values.

$$\epsilon_{\mathrm{eff}}=(\begin{array}{ccc} n_{t}^{2} & 0 & 0\\ 0 & n_{t}^{2} & 0\\ 0 & 0 & n_{l}^{2} \end{array})$$ 4

Here, *n*_t_ and *n*_l_ represent the effective bulk refractive indices of ensembles of uniformly oriented but randomly spatially distributed nanorods with incident light polarized either transverse or longitudinal to the orientation of the rods, respectively (Figure 3a,b). In order to determine these effective refractive indices, the dipole moments in Figure 2b,c were used along with the Clausius–Mossotti relation (eq 2). As expected, the inclusion of rods in the polymer matrix does change the optical properties of the composite, but the strength of the excited dipole clearly has a large impact on the degree of perturbation compared to the corresponding value of the indices of refraction in the pure polymer, where *N* = 1.49 (for PMMA) and *K* = 0. In the case of the transverse excitation, where the dipole is 2 orders of magnitude smaller than the longitudinal case, there is only a slight perturbation from the pure polymer at the location of the dipole shown in Figure 3b. However, in the case of the stronger longitudinal dipole excited in Figure 3a, there is a significant change in *n*_l_ from that of the polymer matrix, and a strong absorption peak is seen in the imaginary part of the index of refraction, *K*_l_, corresponding to the longitudinal LSPR resonance.

**Figure 3:**
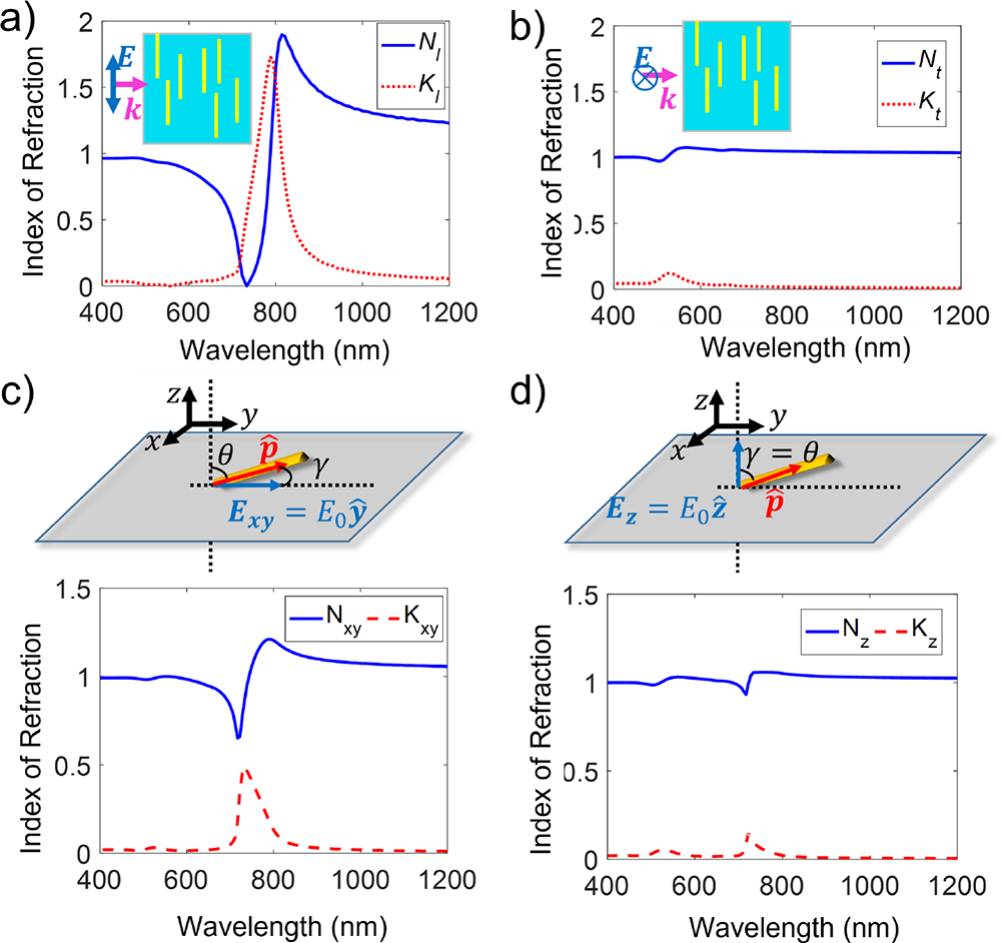
Clausius–Mossotti calculations of indices of refraction based on FDTD simulations of dipole moments of AuNRs. (a) The calculated longitudinal (*N*_l_ and *K*_l_) and (b) transverse (*N*_t_ and *K*_t_) indices of refraction of a hypothetical nanocomposite with the same sized rods (*D* = 12, *L* = 34 nm) all perfectly oriented in the same direction, illuminated with light polarized along (a) and normal (b) to the long axis of rods as schematically illustrated in the insets of each figure. (c) and (d) are calculation results for the in-plane (*N*_*xy*_ and *K*_*xy*_) and out-of-plane (*N*_*z*_ and *K*_*z*_) indices of refraction of a hypothetical thin film, assuming a uniform random distribution in the tilt angles with respect to the normal axis, in the range of 60° < θ < 90° (predominantly in-plane) as schematically shown in the respective insets. *p̂* shows the direction of the main axis of the rod.

For an oblique angle of the incident polarization (γ), the effective index experienced for this system, with uniformly oriented rods, can be calculated by the following mixing formula:^62^

$$\frac{1}{n^{2}}=\frac{\sin^{2}(\gamma )}{n_{l}^{2}}+\frac{\cos^{2}(\gamma )}{n_{t}^{2}}$$ 5

where γ is the angle between the nanorod orientation and the incident polarization. Similar formulas can be written for nanodisks, with the difference that the longitudinal value of the index is expected to be smaller than the transverse value. For more complicated shapes, more generalized forms for the polarizability and index of refraction tensors are required, which is beyond the scope of this work.

#### Calculation of Optical Anisotropy in an Ensemble of Randomly Oriented Particles

In order to determine the optical properties of a composite with a distribution of orientation angles, a new set of angles is needed, corresponding to the oblique angle of individual rods with respect to the plane of a film. The schematics in Figure 3c,d show the details of the model setup, where θ, ϕ, and γ represent the angle between the rod’s long axis (*p̂*) and the vector normal to the film surface (*z* axis), between the *xy*-plane projection of the rod and the *x* axis, and between the *p̂* and the incident electric field vector, respectively. Based on these schematic plots, cos­(γ) = sin­(θ) sin­(ϕ) for the in-plane incident polarization (*y* polarization is assumed since in-plane rod orientation is arbitrary) and γ = θ for out-of-plane incident polarization (*z* polarization).

The effective refractive indices measured by both of these polarizations can then be calculated by extending eq 5, averaging ϕ over 0–180° according to the observed random in-plane rod orientation distributions, and θ over a specific range for tilt angles investigated:

$$\frac{1}{n_{xy}^{2}}=\frac{\left\langle \sin^{2}(\theta )\sin^{2}(\phi )\right\rangle }{n_{l}^{2}}+\frac{1-\langle \sin^{2}(\theta )\sin^{2}(\phi )\rangle }{n_{t}^{2}}=\frac{\left\langle \sin^{2}(\theta )\right\rangle }{2n_{l}^{2}}+\frac{2-\langle \sin^{2}(\theta )\rangle }{2n_{t}^{2}}$$ 6

$$\frac{1}{n_{z}^{2}}=\frac{\left\langle \cos^{2}(\theta )\right\rangle }{n_{l}^{2}}+\frac{\left\langle \sin^{2}(\theta )\right\rangle }{n_{t}^{2}}$$ 7

An example calculation for an ensemble of identically sized but randomly distributed and randomly oriented AuNRs is shown in Figure 3c,d. The orientational distribution of the AuNRs follows the description of the composite described in Figure 2a, with a random orientation distribution in the in-plane direction, and a uniform out-of-plane orientation distribution confined in the tilt angle range of 60° < θ < 90°. The background index was assumed to be *N* = 1 and *K* = 0. Similar calculations for other in-plane and out-of-plane distributions are shown in SI Figures S8 and S9, respectively. Compared with the ensemble with AuNRs showing a unidirectional orientation distribution as described in Figure 3a,b, the effective refractive index spectra in Figure 3c,d include features of both the longitudinal and the transverse LSPR resonances. The relative strength of a particular resonance under the two excitation polarizations can describe the difference in the orientational distribution in the two respective directions, noting that the longitudinal LSPR is much stronger, and is thus seen clearly in both *n*_*xy*_ and *n*_*z*_ values.

### Calculation of the Orientation Order Parameter

SE measurements contain phase information on the reflected light, as shown in eq 1, regardless of the reflected intensity of light. As such, measurements of the plasmonic response of NPs can be made even at extremely low intensities where the majority of the signal originates from plasmonic NP scattering and in nanometer-sized films, as shown in Figure 1d. To evaluate the average orientation of AuNRs in VASE experiments and compare the results with simulations, the orientation order parameter *S*_*z*_, with respect to the normal axis of the film, can be used. *S*_*z*_ is defined as the ensemble average of the out-of-plane tilt distribution of asymmetric dipolar objects (liquid crystalline molecules or nanorods and nanodisks here) as follows:^63,64^

$$S_{z}=\frac{3\langle \cos^{2}(\theta )\rangle -1}{2}=\frac{K_{z}-K_{xy}}{K_{z}+2K_{xy}}$$ 8

As shown in this equation, *S*_*z*_ can be directly measured using measurements of optical birefringence, where *K*_*z*_ and *K*_*xy*_ are the imaginary components of the calculated out-of-plane and in-plane indices of refraction at the corresponding dipole resonance, respectively (inset of Figure 1d). As such, *S*_*z*_ is the main outcome of VASE experiments that can be compared with the corresponding modeled values, calculated directly based on the assumed distributions of NPs (as shown for example for the calculated distributions in Figures S8 and S9). The minimum value of *S*_*z*_ = −0.5 represents the case where all AuNRs are fully in-plane, while the maximum of *S*_*z*_ = 1 represents the case where all AuNRs have a fully out-of-plane alignment. *S*_*z*_ = 0 indicates a completely random distribution of orientation angles with no preference for in-plane or out-of-plane orientations.

## Results and Discussions

### The Dependence of the Orientation Order Parameter on the Distribution of Particle Orientations

Figure 4a shows how the calculated values of the orientation order parameter *S*_*z*_ depend on the assumed distributions of angles of AuNRs in a hypothetical thin film. Two types of distributions are assumed here. For predominantly in-plane AuNRs, a uniform distribution is assumed in the range of θ_min_ < θ < 90°, and the figure is plotted as a function of the minimum allowed tilt angle (θ_min_). For predominantly out-of-plane distribution, the opposite is assumed where the distribution covers a range of 0° < θ < θ_max_ and the orientation order parameter is plotted vs the maximum allowed tilt angle (θ_max_). A detailed version of these distributions can be found in Figure S14 of the Supporting Information. In both cases, a clear trend is observed toward *S*_*z*_ = 0 when all angles are allowed to have the same weight, θ_min_ = 0° or θ_max_ = 90°, respectively. For θ_min_ = 90°, indicating fully in-plane alignment as rods are restricted to lie in-plane, *S*_*z*_ = −0.5. The reverse is true for θ_max_ = 0°, where rods are restricted to be fully out-of-plane and *S*_*z*_ = 1.

**Figure 4:**
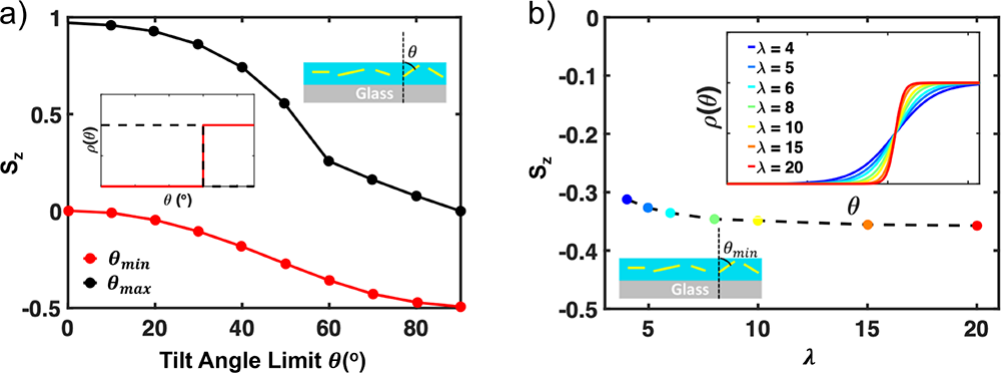
(a) The calculated orientation order parameter (*S*_*z*_) bluebased on simulations vs the assumed minimum tilt angle limit (θ_min_, red) in mostly in-plane AuNRs or assumed maximum tilt angle limit (θ_max_, black) in mostly out-of-plane AuNRs. In each case, the tilt angle is assumed to be random within the window with a uniform distribution in a range of either θ_min_ < θ < 90° (red, in-plane) or 0° < θ < θ_max_ (black, out-of-plane), respectively. The shapes of these uniform distributions, as sharp step functions, are shown in the inset, with corresponding colors. (b) The calculated orientation order parameter (*S*_*z*_) for distributions of mostly in-plane AuNRs around a cutoff of θ_min_ = 60°, where a smooth step function is used with variable λ. Decreasing λ broadens the distribution to include more out-of-plane AuNRs with smaller values of the tilt angle θ.

This uniform distribution with a cutoff in the tilt angle is useful when estimating the range of angles in experimental systems where *S*_*z*_ is determined based on the birefringence of the index of refraction. However, in the experimental systems, one cannot assume the distribution is perfectly flat with all tilt angles having equal probability. While there may be a driving force for a large portion of AuNRs to be below or above a certain angular cutoff, either due to confinement of the film thickness (Figure 4a) or order factors such as block copolymer self-assembly,^14,16^ a smaller population might still exist outside the cutoff region, due to size heterogeneity or kinetic restraints. It is therefore incumbent to estimate the robustness of the estimated values of *S*_*z*_ if a broader distribution is considered. In order to investigate the sensitivity of *S*_*z*_ to the cutoff value, the width of the distribution around the target value was varied. Figure 4b shows an example of such calculations for a predominantly in-plane distribution with a cutoff value of θ_min_ = 60°. Here, the parameter λ is used to control the sharpness of the cutoff in the probability distribution function for the rod orientation as

$$p(\theta )=\frac{1}{2}[1+\tanh\lambda (\theta -\theta_{\min})]$$ 9

θ_min_ = 60° was selected here because it is expected that the largest error for θ_min_ (or θ_max_ for out-of-plan orientation) to occur away from the extremes of *S*_*z*_ = −0.5, *S*_*z*_ = 1 or *S*_*z*_ = 0, where *S*_*z*_ varies more strongly with θ.

As seen in the inset of Figure 4b at low values of λ, a significant population of oriented AuNRs can adopt orientations below the angular cutoff (more out-of-plane), while increasing λ produces sharper distributions, analogous to what was measured using a step function. Figure 4b shows that even at reasonably low values of λ, there is only a slight increase in the value of the orientation order parameter (by less than 0.05). This slight increase in the orientation order parameter is reasonable, given that decreasing λ increases the population of AuNRs with smaller angles, thus increasing the out-of-plane population. The measured shift in *S*_*z*_ is analogous to a change in θ_min_ from θ_min_ = 60° to θ_min_ = 50° when compared to the corresponding data in Figure 4a, which represents this additional out-of-plane population. This analogy demonstrates the robustness of this simple approximation of using a flat population with a sharp cutoff.

### The Dependence of Orientation Order Parameter on Size Heterogeneity

The assumption that the optical response from a single rod can be used to extrapolate that of a real composite only applies if all of the nanorods in the composite have the same size and aspect ratios. As such, one needs to explore the robustness of the calculated *S*_*z*_ to NP size heterogeneity. To do so, FDTD calculations were extended to AuNRs with a variety of lengths and diameters to obtain their optical behavior (details in the methods section, the SI, and Figures S3–S7). To mimic an experimental system, the calculations of the effective-medium index of refraction were performed, making similar assumptions as before (low density of AuNRs, dipole approximation, etc.), along with an assumed Gaussian distribution of rod diameters and lengths. For simplicity, first, the average dipole moment of the distribution of AuNRs was calculated (Figure S4) as detailed in the methods section and SI. For both directions of polarization, the modeled dipole moments for various degrees of size heterogeneity have similar LSPR resonances with reasonably similar intensity as the corresponding data for single-sized AuNR (shown in Figure 2b,c). However, the spectra are broadened with increased heterogeneity­(Figure S4), as is typically observed in experimental measurements.

Using the calculated average dipole moments of heterogeneously sized AuNRs, the calculations of the indices of refraction and *S*_*z*_ can be readily extended to these systems, as shown in Figures S5 and S6. Figure 5a,b show the calculated longitudinal (*n*_l_) and transverse (*n*_t_) indices of refraction for the most polydisperse system (the broadest Gaussian distribution of sizes), respectively. The comparison between this data and data performed on monodisperse AuNRs (Figure 3a,b, respectively) shows reasonable similarity between the indices of refraction. In particular, due to the relatively weak transverse dipolar response, *n*_t_ is largely unchanged between the polydisperse and the monodisperse systems. However, some noticeable differences are observed in *n*_l_ when the data in Figure 5a is compared with the corresponding data in Figure 3a. In the measured *N*_l_, the perturbation from the polydisperse AuNRs occurs over a broader range of wavelengths, with a noticeable shoulder at lower wavelength values (also seen as the trend in increasing heterogeneity in Figure S6a). The LSPR resonance as seen in *K*_l_ is also noticeably broader (Figure S6b), indicating that the width of the peak can be used as an experimental measure of size heterogeneity.

**Figure 5:**
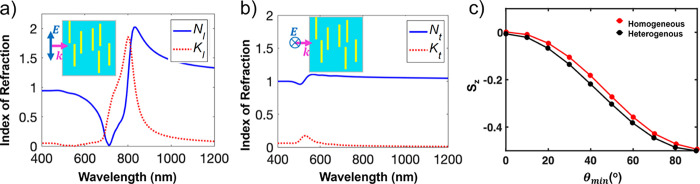
Clausius–Mossotti calculations of indices of refraction based on FDTD simulations of dipole moments of heterogeneously sized AuNRs. (a) The calculated longitudinal (*N*_l_ and *K*_l_) and (b) transverse (*N*_t_ and *K*_t_) indices of refraction of a hypothetical bulk nanocomposite with a Gaussian distribution of NP sizes and diameters all perfectly oriented in the same direction, illuminated with light polarized along (a) and normal (b) to the long axis of rods as schematically illustrated in the insets. (c) The calculated orientation order parameter vs θ_min_ for predominantly in-plane AuNRs (θ_min_ < θ < 90°), for monodisperse particles (homogeneous, red) and particles with a Gaussian distribution of lengths and diameters (heterogeneous, black).

Using these calculations, as before, the orientation order parameter was calculated for various distributions of AuNR orientations and compared with those of the monodisperse distribution. Figure 5c compares the calculated values of *S*_*z*_ for predominantly in-plane orientations for polydisperse (black) and monodisperse (red) distributions. As seen in this figure, the orientation order parameter is reasonably insensitive to size heterogeneity, as it is calculated based on the value and strength of the LSPR resonance. The effect of size heterogeneity is predominantly observed in the breadth of the resonance, while the orientation is measured through relative amplitude (birefringence), decoupling the two quantities. This is a unique feature arising in optical birefringence measurements that allows a separate estimation of the degree of monodispersity of the NPs, through the width of the resonance, and the orientation orderparameter through the optical birefringence.

However, it is worth noting that the calculated orientation order parameter is consistently lower in polydisperse systems, as the distribution includes longer rods with stronger longitudinal dipole moments, thus systematically generating stronger birefringence, reducing the orientation order parameter. In contrast, while the effect of the inclusion of smaller AuNRs is seen more strongly in the blue-shifted shoulder of the extinction spectrum (*K*), the smaller cross-section of these AuNRs generates a negligible effect in increasing the value of the orientation order parameter. However, the overall change in the orientation order parameter due to NP size heterogeneity is small and mostly within the same range of uncertainty (<0.05 in variations) as it was when assuming a sharp cutoff in the distribution of angles, and thus within the expected experimental uncertainty.

### The Effect of Particle Shape on the Orientation Order Parameter

The method described here can be readily extended to PNCs with other types of inclusions with uniaxial symmetry, as long as the size of the particles is much smaller than the wavelength of the incident light, their number density is low, and the particles are reasonably well-dispersed. For example, Figure 6 shows the orientation order parameter calculations for a system of gold nanodisks (AuNDs). For this system, since the major axis of the particle is normal to its plane, a predominantly in-plane distribution is described with small values of θ. As such, increasing the cutoff angle increases the orientation order parameter toward *S*_*z*_ = 0, while the opposite is true for AuNRs. Figure 6c shows the calculated orientation order parameters for in-plane distributions of both AuNRs and AuNDs, spanning a range of −0.5 < *S*_*z*_ < 0 in both cases. It also holds that the nanodisk results are robust to distribution in angles around the cutoff as well as to polydispersity in nanodisk shape. It is important to note that changing the definition of the main axis for the nanodisk will not produce an identical dependence of *S*_*z*_ on the angle, as there would be two longitudinal components with large dipole moments in eq 4, necessitating a change in eq 5. As such, it is more convenient to choose the primary axis of asymmetry for defining the angle θ as it is defined here.

**Figure 6:**
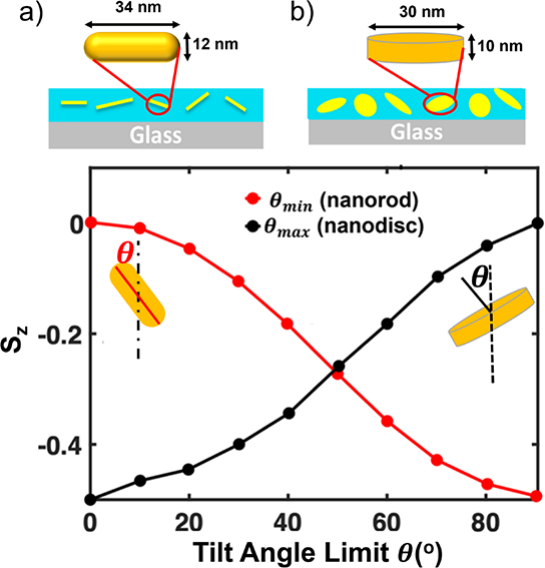
(a and b) Schematic representations of polydisperse gold nanorods (a) and gold nanodisks (b) in PNC thin films. The chosen values of the average length and diameters are based on experimental values. (c) Calculated orientation order parameter vs the assumed minimum tilt angle limit (θ_min_, red) in predominantly in-plane AuNRs or assumed maximum tilt angle limit (θ_max_, black) for predominantly in-plane AuNDs. In each case, the assumed distribution is random in a range of tilt angles either between θ_min_ < θ < 90° (red, AuNR) or 0° < θ < θ_max_ (black, AuND), respectively. The insets schematically show the definition of tilt angles for each case.

### Evaluating the Validity of the Approach in Simulations

The simulated data presented in the previous sections demonstrate that the calculated orientation order parameter is robust with respect to variations in the detailed shape of the angular distributions as well as nanoparticle heterogeneity. While it is impossible to invert a measured value of *S*_*z*_ to a unique distribution of angles, this approach can be validated by estimating the margin of error in heterogeneous systems. To this extent, both simulations and experiments can be used as a means for validation.

Focusing on simulated data, for example for distribution with θ_min_ = 60° (60° < θ < 90°), the theoretically calculated *S*_*z*_ for monodispersed AuNRs is *S*_*z*_ = −0.38, increasing to *S*_*z*_ = −0.36 for the polydispersed AuNRs (Figure 5c). Mimicking the SE experiments, *S*_*z*_ can be obtained for the monodispersed AuNRs based on the calculated *K*_*xy*_ and *K*_*z*_ shown in Figure 3c,d, respectively. The mimicked “experimental” *S*_*z*_(SE) in this case is *S*_*Z*_(SE) = −0.37 ± 0.05, with the error representing the choice made based on the width of the resonance. Similar calculations, based on mimicked experimental extinction for θ_min_ = 30° (data shown in Figure S8) result in *S*_*z*_(SE) = −0.08 ± 0.05, which corresponds to θ_min_(SE)= 26° ± 5° for monodispersed AuNRs. These values are the same within the error. The origins of these errors lie in the resolution of calculated spectra and the assumption that the signal is from point dipoles as opposed to AuNRs with finite width, which have broadened spectra. Similar calculations for the experimentally mimicked *S*_*Z*_(SE) of out-of-plane AuNRs yield the same values of (θ_max_) within error, as those used to calculate *S*_*z*_ (Figure S9). The data for θ_min_ = 0° and θ_min_ = 90° are trivial, as the longitudinal LSPR resonance is only seen either in the *K*_*xy*_ (θ_min_ = 0°) or *K*_*z*_ directions (θ_min_ = 90°, Figure S8) directions, respectively.

### Experimental Validation of VASE Measurements of the Orientation Order Parameter

To validate the VASE measurements of the orientation order parameter, several experimental systems were explored with increasing complexity. Specifically, we looked at the cases of spherical NPs (i.e., *S*_*z*_ = 0), AuNRs confined in-plane (i.e., *S*_*z*_(SE) = −0.5), AuNRs in thin films, and, finally, AuNRs in films of various thicknesses.

When spherical NPs are used, orientation order parameter is expected to be *S*_*z*_ = 0, as the objects are isotropic. As seen in Figure 7a, 10 nm Au spheres are well dispersed and isotropic, as per the SEM image. Despite their inherent size heterogeneity, these spheres are presumed to always have isotropic orientation. Figure 7b shows the measured in-plane (*K*_*xy*_) and out-of-plane (*K*_*z*_) extinction spectra when the VASE data is force-fitted to an anisotropic model. The resultant orientation order parameter of *S*_*z*_(SE) = −0.08. However, we note that the error in the fitting between using isotropic and anisotropic models is negligible, meaning that the data in Figure 7b represents an overfitted condition. As such, the result can be used as an empirical approach to estimate the inherent error of determining *S*_*z*_ using VASE experiments.

**Figure 7:**
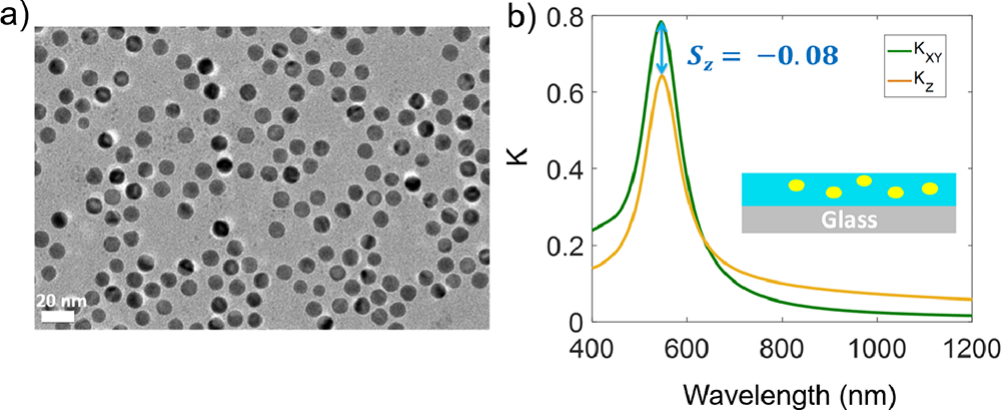
Orientation order parameter calculations for isotropic particles. (a) SEM image of 10 nm Au spheres dispersed in a PS film matrix. While the spheres have some degree of size heterogeneity, they can be presumed to always have isotropic orientation. (b) The measured in-plane (*K*_*xy*_) and out-of-plane (*K*_*z*_) extinction spectra when VASE data is force-fitted to an anisotropic model. Using anisotropy does not improve the fitting and results in a measured orientation order parameter of *S*_*z*_(SE) = −0.08. This value can be considered as the inherent error of determining *S*_*z*_ using VASE experiments.

In the other limit, the maximum in-plane orientation can be achieved in a film without a polymer matrix, where all AuNRs are forced to be fully in-plane. As shown in Figure S13, in this case, the orientation order parameter is measured to be *S*_*z*_(SE) = −0.5, indicating a perfect in-plane AuNR orientation, as expected. Interestingly, in this example, AuNRs are moderately linked end-to-end, resulting in significant red-shifting and broadening of the LSPR resonance. Despite this linking and resultant spectral change, the measured *S*_*z*_(SE) remains unaffected and matches expected outcomes. This is true in the case of side-by-side aggregation as well (Figure S12). In both cases, the polymer brush provides a reasonable distance between the AuNRs, preventing them from physically touching each other or producing strong hot spots, limiting the effect on the birefringence. This is a strong validation for measurements of optical birefringence as a powerful technique for determining the orientational distribution of elongated objects.

As mentioned earlier, it is incredibly difficult to define the out-of-plane distribution of AuNRs in experimental systems on large enough ensembles to directly validate this approach experimentally. It is, however, possible to estimate the extent of the error in experimental systems under some conditions. For example, as seen in Figure 1d, the experimentally measured value of *S*_*z*_(SE) for mostly in-plane AuNRs, in a 12 ± 2 nm film (roughly the same thickness as the average AuNR diameter) is *S*_*z*_(SE) = −0.44 ± 0.05. Using the data in Figure 5c for heterogeneous rods, θ_min_ = 68° ± 5°. AuNRs with lengths between 34 nm < *L* < 38 nm in a 12 nm film can assume theoretical θ_min_ tilt angles in the range of 67°–73°, at their most extreme out-of-plane states. As such, a distribution of angles between 68° < θ < 90° is an experimentally reasonable outcome for this data. When polymers with different molecular weights are used for the matrix polymers, films of the same thickness, including the same type of grafted NPs show different values of the orientation order parameter. This is consistent with the observation that for larger molecular weights, the AuNRs are tilted more in-plane (Figure S11). One generally expects more in-plane order in higher molecular weight PNCs, as they can kinetically trap NPs in the in-plane direction. This is indeed the case for PS grafted AuNRs in PMMA matrix, as shown in Figure S11 for films with thicknesses slightly higher than the AuNR length.

As seen in Figure 8, when the film thickness is varied in PNCs with well-distributed AuNRs (PS graft in PS matrix of the same molecular weight), the orientation order parameter changes from in-plane (*S*_*z*_(SE) = −0.34 ± 0.05, for *h* = 40 nm) to slightly out-of-plane (*S*_*z*_(SE) = 0.08 ± 0.05 for *h* = 107 nm) when the film thickness is increased. Using data in Figure 4a, we can estimate a tilt angle distribution 58° < θ < 90° (θ_min_ = 58° ± 5°) in the *h* = 40 nm film, while the corresponding distribution in the *h* = 107 nm film is 0° < θ < 78° (θ_max_ = 78° ± 5°), corresponding to nearly isotropic distribution with slight elimination of very large angles. This means that perfectly in-plane rods are not favored in thick films.

**Figure 8:**
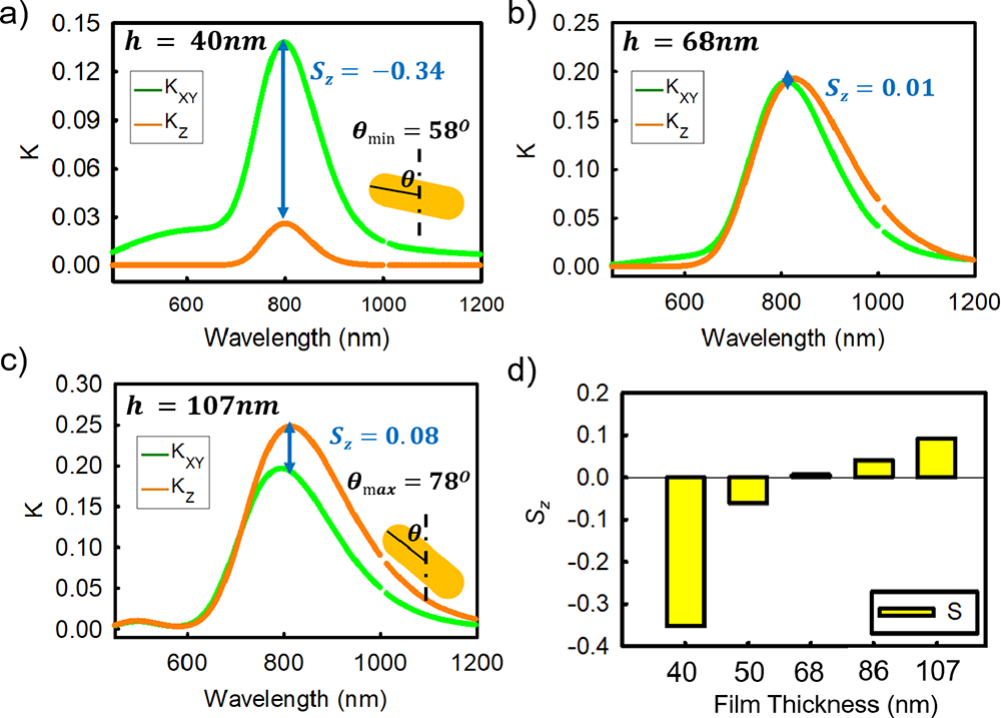
(a–c) The measured in-plane (*K*_*xy*_) and out-of-plane (*K*_*z*_) extinction coefficients vs film thickness for AuNRs with 20 kg/mol PS brush in 20 kg/mol PS matrix. The measured film thicknesses and the orientation order parameters are shown in the inset of each figure: (a) *h* = 40 ± 2 nm, *S*_*z*_(SE) = −0.34 ± 0.05 (b) *h* = 68 ± 2 nm, *S*_*z*_(SE) = 0.01 ± 0.05, and (c) *h* = 107 ± 2 nm, *S*_*z*_(SE) = −0.08 ± 0.05, respectively. (d) The dependence of *S*_*z*_(SE) on film thickness for a number of measured samples.

## Conclusions

We propose an effective optical medium approach to model the tilt orientation order parameter of axially symmetric gold nanoparticles embedded in a polymer matrix with effective optical anisotropy. In particular, the nanocomposite was treated as a bulk metamaterial with an effective uniaxial birefringence due to the preferred in-plane nanorod or nanodisk orientation. FDTD calculations of NPs with realistic size, shape, and size heterogeneity were used to calculate the induced dipole-moment of each type of inclusion, and then used to calculate the effective complex, birefringent index of refraction of the medium, using dipole approximation. We calculate the orientation order parameter under various conditions and demonstrate that it is robust to shape heterogeneity and small variations in the distribution of orientation angles.

Spectroscopic ellipsometry was then used to directly measure the orientation order parameter and the degree of aggregation in PNCs, including heterogeneous distribution of nanorods and nanospheres, to demonstrate the feasibility of this approach in characterizing the average tilt angle and the degree of aggregation of nanoparticles in polymer nanocomposites, in a robust and predictable manner. The combinations of the theoretical predictions and experimental measurements in PNCs with various film thicknesses and properties demonstrate the unique capability of using spectroscopic ellipsometry as a quantitative and nondestructive method to characterize ensemble-level properties of polymer nanocomposites, opening the opportunity to gain structure/property relationships even in nanometer-sized thin films.

## Supplementary Material



## Notes

Simulation details, calculations of the dipole moment based on simulations, details of nanorod synthesis, and additional experimental details (PDF)

## ∥

KLA Corporation, Hillsboro, Oregon 97006, United States

## ⊥

Department of Materials Science and Engineering, Massachusetts Institute of Technology, Cambridge, Massachusetts 02139, United States.

## #

Dow Inc., Midland, Michigan 48674, United States.
