# Supplementary material for: The Effect of Nanoparticle Shape, Orientation, and Heterogeneity on the Optical Birefringence of Polymer Nanocomposites
Source: J Phys Chem C Nanomater Interfaces. 2026 Feb 16;130(8):3005–15. doi: 10.1021/acs.jpcc.5c07736 (PMC12951575; doi:10.1021/acs.jpcc.5c07736)
Supplement: Supplementary file 1 [file jp5c07736_si_001.pdf]

# Supporting Information: The Effect of Nanoparticle Shape, Orientation, and Heterogeneity on the Optical Birefringence of Polymer Nanocomposites

Chen Li,[†,‡,#] Sathya Edamadaka,[†,¶,#] Ethan Glor,[†,§] Melissa J. Vettelson,[†] Nathaniel S Watkins,[†] Jacob Faber-Rico,[†] Russell J. Composto,[‖] Robert C. Ferrier, Jr.,[*,⊥] and Zahra Fakhraai[*,†]

†*Department of Chemistry, University of Pennsylvania, Philadelphia, Pennsylvania 19104, United States*

‡*Present Location: KLA Corporation, Hillsboro, Oregon 97006, United States*

¶*Present Location: Department of Materials Science and Engineering, Massachusetts Institute of Technology, Cambridge, Massachusetts 02139, United States*

§*Present Location: Dow Inc., Midland, Michigan, 48674, United States*

‖*Department of Materials Science and Engineering, University of Pennsylvania, Philadelphia, Pennsylvania 19104, United States*

⊥*Department of Chemical Engineering and Materials Science, Michigan State University, East Lansing, Michigan 48824, , United States*

#*Contributed equally to this work*

E-mail: ferrier5@msu.edu; fakhraai@sas.upenn.edu

## Near-field Multipole Expansion

Under optical illumination, a plasmonic nanoparticle can be assumed to be a passive radiation source, whose scattered electric near-field ($\mathbf{E}$) can be described with the vector ($\mathbf{A}$) and scalar ($\phi$) potentials, assuming $e^{-i\omega t}$ time conventions:

$$\mathbf{E} = -\nabla\phi + i\omega\mathbf{A} \tag{1}$$

These potentials are related to the radiation sources through the Helmholtz integrals:[1]

$$\phi(\mathbf{r}) = \frac{1}{\epsilon}\int \rho(\mathbf{r}')\frac{e^{ik|\mathbf{r}-\mathbf{r}'|}}{4\pi|\mathbf{r}-\mathbf{r}'|}dV' \tag{2}$$

$$\mathbf{A}(\mathbf{r}) = \mu\int \mathbf{J}(\mathbf{r}')\frac{e^{ik|\mathbf{r}-\mathbf{r}'|}}{4\pi|\mathbf{r}-\mathbf{r}'|}dV' \tag{3}$$

where $\rho(\mathbf{r}')$ and $\mathbf{J}(\mathbf{r}')$ are the charge density and current density distribution functions in the source region $V'$, respectively. As only the far-field response is of interest here, the nanoparticle (NP) scattering object can be viewed as a point-like radiation source. Thus, by assuming $\mathbf{r}' \ll \mathbf{r}$, the potentials can be Taylor expanded into multipole scattering modes around $\mathbf{r}'$.[1,2] For scattering objects much smaller than the wavelength ($\lambda$) with simple shapes (such as spheres or rods), we can use the approximation that the dipole mode dominates the far-field response, ignoring higher-order multipole modes. As such, we can keep the leading term in the expansion only:

$$\phi(\mathbf{r}) = -\frac{1}{\epsilon}\mathbf{p}\cdot\nabla G \tag{4}$$

$$\mathbf{A}(\mathbf{r}) = -i\omega\mu\mathbf{p}\nabla G \tag{5}$$

where $G = e^{ikr}/r$ is the scalar Green's Function, and $\mathbf{p}$ is the excited dipole moment that can be calculated from the near-field current distribution by applying the continuity equation:[1,2]

$$\mathbf{p} = \int \rho(\mathbf{r}')\mathbf{r}' dV' = \frac{i}{\omega} \int \mathbf{J}(\mathbf{r}') dV' \tag{6}$$

Since measurable properties such as scattering or extinction spectra should be modeled as source-free responses of the optical excitation, the radiating current above refers to the polarization current, defined as the time derivative of electric polarization:

$$\mathbf{J} = \frac{\partial \mathbf{P}}{\partial t} = -i\omega P \tag{7}$$

The excited dipole moment is thus related to the local electric near-field distribution by the following equation:

$$\mathbf{p} = \int \mathbf{P}(\mathbf{r}') dV' = \int (n^2(\mathbf{r}') - n_b^2)\mathbf{E}(\mathbf{r}') dV' \tag{8}$$

where $n$ and $n_b$ are the local and the background refractive indices, respectively.

## Simulation Details

Lumerical FDTD software was used for finite difference time domain (FDTD) simulations to calculate the dipole moments of model gold nanorods (AuNRs) and nanodiscs (AuNDs). Nanorods (Figure S1) were modeled as cylinders with spherical ends. Nanodiscs (Figure S2) were modeled as short cylinders with their main axis along the length of the cylinder. Each structure was separately modeled by exciting with a built-in 1.33 fs broadband total-field scatter-field (TFSF) source polarized either parallel or perpendicular to the rod's long axis (or cylinder's main axis), referred to as "longitudinal" and "transverse" polarizations, respectively. A background refractive index of 1.49 was used to model a typical polymer's index of refraction. The dielectric constant of gold and its spectral dispersion were obtained from the CRC Handbook of Chemistry & Physics.[3] Perfectly matched layer (PML) boundary

conditions were assumed to simulate the semi-infinite PMMA matrix. Other details of these simulations were kept the same as our previously reported calculations.[4] Figure S1a shows an example of the simulation box layout. Figure S1b and S1c show the calculated extinction cross-section of the prototypical AuNR with $L = 34$ nm and $D = 12$ nm, which matches the experimentally measured extinction coefficients based on UV-visible spectroscopy (Figure 1 of the main text, for example). The calculated dipole moments for this AuNR are shown in Figure 1 of the main text. Figure S2 shows the corresponding dipole moments for a nanodisc with $L = 30$ nm and $D = 10$ nm.

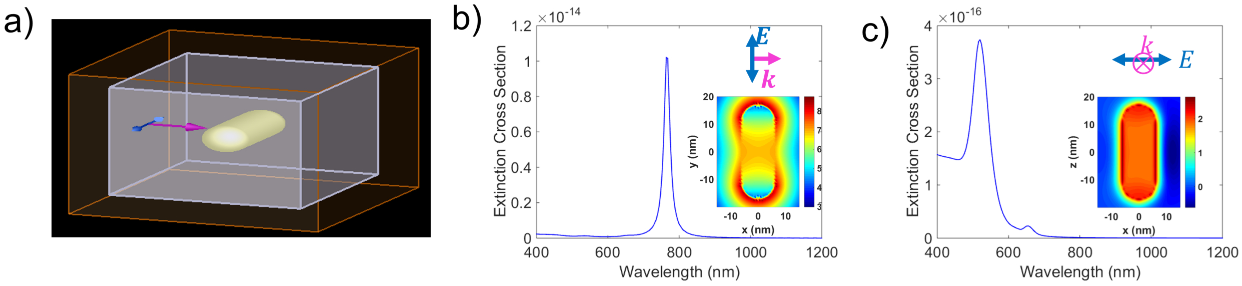


Figure S1: (a) Representative image of the simulation box used for calculations of extinction coefficients. The yellow square box shows the simulation region, and the gray area shows the boundaries of the TSFS source, within which the scattering field is calculated. The example shown here is the longitudinal illumination of an AuNR with $L = 34$ nm and $D = 12$ nm. (b, c) The calculated extinction cross-section for this AuNR, when illuminated in the longitudinal (b) and transverse (c) directions. The calculated dipole moments are shown in Figure 1 of the main text.

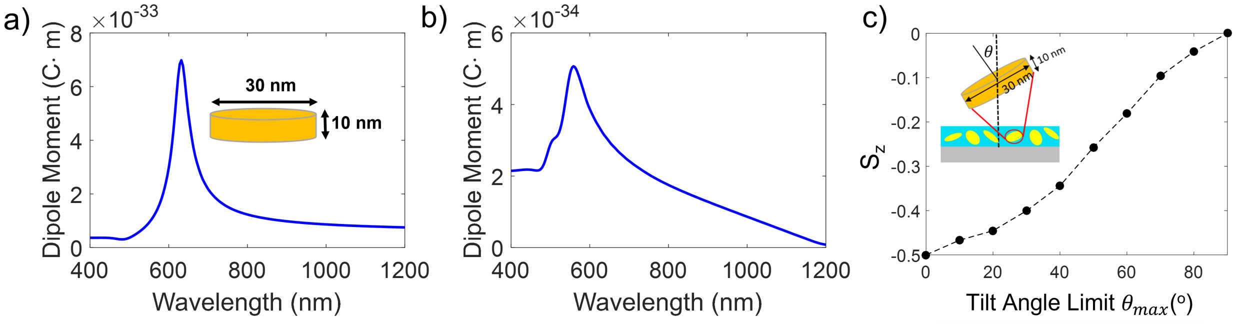


Figure S2: (a,b) The calculated dipole moment of a PNC containing nanodiscs for (a) longitudinal and (b) transverse polarization of the incident light. The inset in (a) shows the diameter ($D = 30$ nm) and thickness ($L = 10$ nm) of the disc. (c) The calculated orientation order parameter ($S_z$) for uniform distribution of angles, between $0 < \theta < \theta_{max}$ vs. maximum tilt angle limit ($\theta_{max}$). The inset shows the geometry of the disc with respect to the tilt angle $\theta$.

**Table S1: Detailed list of simulated AuNR size and aspect ratios**

| AuNR Diameter ($D$) | AuNR Length ($L$) | Aspect Ratio ($\frac{L}{D}$) | LSPR Wavelegnth ($nm$) |
|---|---|---|---|
| 14 | 42 | 3 | 780.76 |
| 14 | 41.4 | 2.957 | 775.57 |
| 14 | 41 | 2.929 | 774.53 |
| 13 | 38 | 2.923 | 774.22 |
| 12 | 35 | 2.917 | 774.83 |
| 14 | 40.7 | 2.907 | 769.30 |
| 14 | 40.2 | 2.871 | 767.31 |
| 14 | 40 | 2.857 | 762.07 |
| 12 | 34 | 2.833 | 768.30 |
| 14 | 39.3 | 2.807 | 760.15 |
| 14 | 39 | 2.786 | 756.16 |
| 13 | 36 | 2.769 | 750.56 |
| 12 | 33 | 2.75 | 756.16 |
| 14 | 38.5 | 2.75 | 750.25 |
| 14 | 38 | 2.714 | 745.64 |
| 14 | 37.6 | 2.686 | 744.34 |
| 14 | 37 | 2.643 | 739.23 |
| 14 | 36.8 | 2.629 | 738.04 |
| 14 | 36.5 | 2.607 | 733.13 |
| 14 | 36 | 2.571 | 727.52 |

To model the optical properties of a non-ideal composite with heterogeneous NR sizes, the same simulations were repeated for a range of 20 AuNRs with lengths ($L$) and diameters ($D$) in the range of 33 nm< $L$ <42 nm and 12 nm< $D$ <14 nm, respectively (aspect ratios between blue2.5 $< L/D <$ 3, as shown in Table S1. These values were chosen to roughly mimic the size distribution of AuNRs in solution, obtained from scanning electron microscopy (SEM) analysis of the dried AuNRs, as previously reported. Using the method described above the individual dipole moments of each AuNR, for the longitudinal and transverse directions were calculated, accordingly. Figure S3 shows the calculated longitudinal and transverse dipole moments for these AuNRs. As seen in this figure, increasing AuNR length and decreasing the diameter, both result in increasing resonance wavelength. As such, the aspect ratio $L/D$ can be used as a convenient figure of merit for random sampling of sizes to calculate the effect of size heterogeneity on LSPR. We also note that the dipole moment

depends on the total volume of gold, which increases linearly with $L$. As such larger AuNRs generally contribute more to the signal. As such, fewer smaller particles were considered for weighting, more centered around the target LSPR resonance.

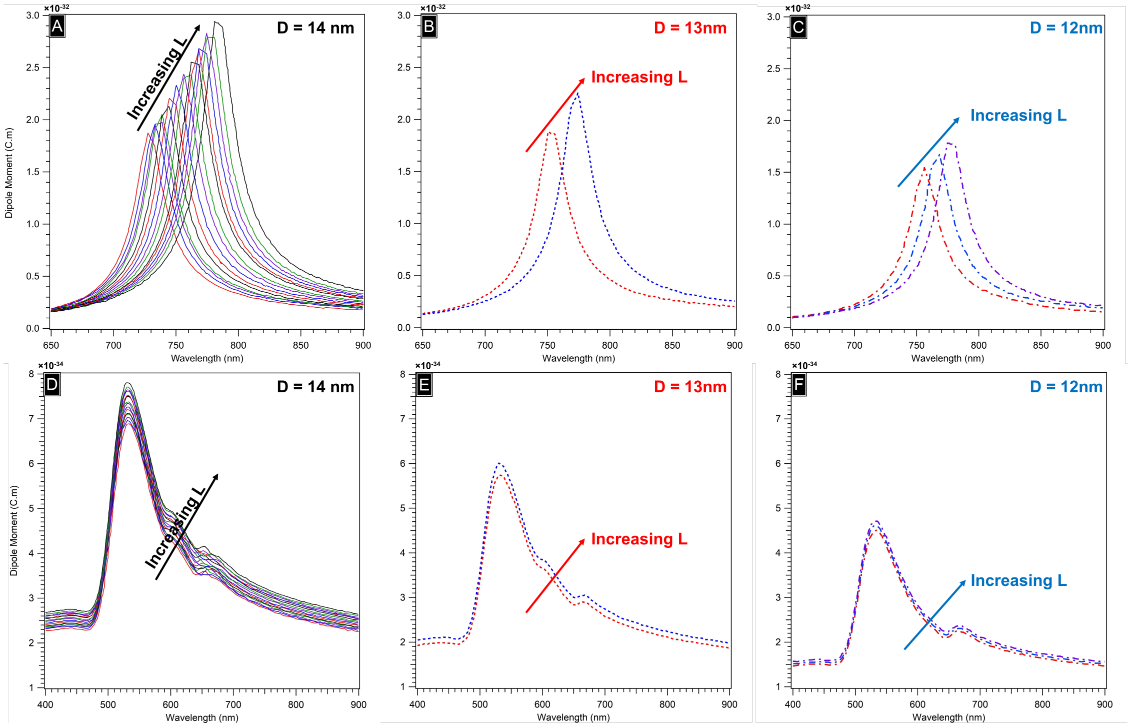


Figure S3: Calculated dipole moments for the (a-c) longitudinal and (d-f) transverse polarization of the incident light, for AuNRs with $D = 14$ nm, and 36 nm< $L$ <42 nm (black arrow), $D = 13$ nm, 36 nm< $L$ <36 nm (red arrow), and $D = 12$ nm, 33 nm< $L$ <35 nm (blue arrow). The corresponding LSPR values are reported in Table S1.

To combine these individual simulation results to model PNCs with varying degrees of heterogeneity, a normal distribution of AuNR aspect ratios was considered. To determine the total dipole moment of a given sample, a convex combination of individual AuNR dipole moments was formed with weights given by the Gaussian distributions (example shown in Figure S7). Figures S4-S6 illustrate how including nanorods with increasing degrees of variations in their length and diameter (increasing the width of the Gaussian distribution) affect the calculated dipole moment and the effective-medium indices of refraction in the longitudinal and transverse polarization of the incident light. As seen in these figures, when a sharp distribution is assumed, only including the $D = 12$ nm and $L = 34$ nm AuNR the

LSPR and the calculated extinction spectra ($K_t$ and $K_l$) are sharp. As increasingly more AuNR sizes are included, by increasing the width of the Gaussian probability function, the spectra become broadened and show shoulders. The broadest spectra (also shown in Figure 5 of the main page) were observed when all 20 calculated dipole moments shown in Table S1 were averaged with their corresponding weight shown in Figure S7.

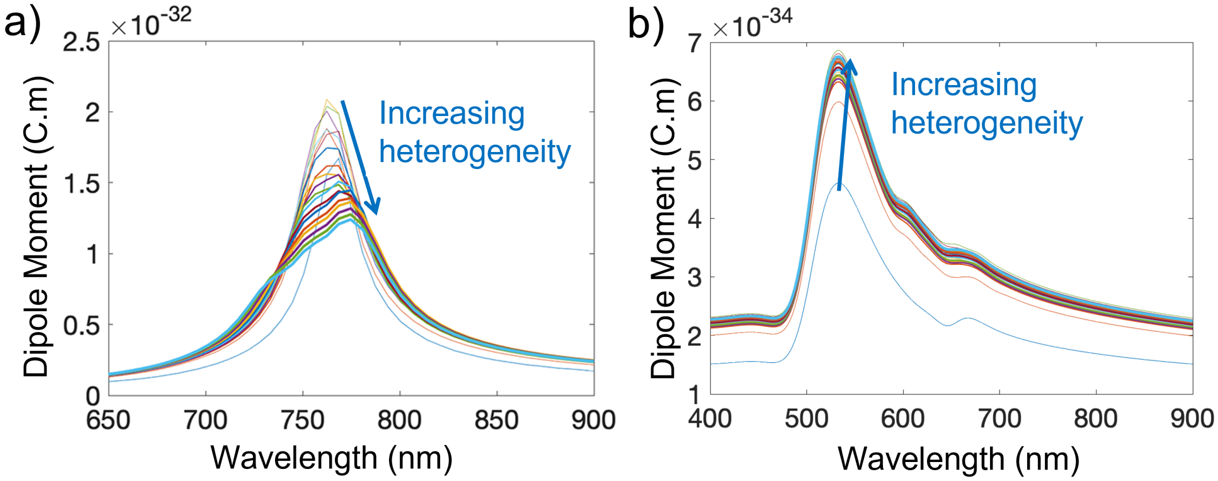


Figure S4: Dipole moments calculated for (a) parallel and (b) transverse polarization of the incident light, for composites with varying degrees of size heterogeneity as detailed in the text. The blue arrows show the direction of increased heterogeneity, where a broader range of size distributions is included in the calculations.

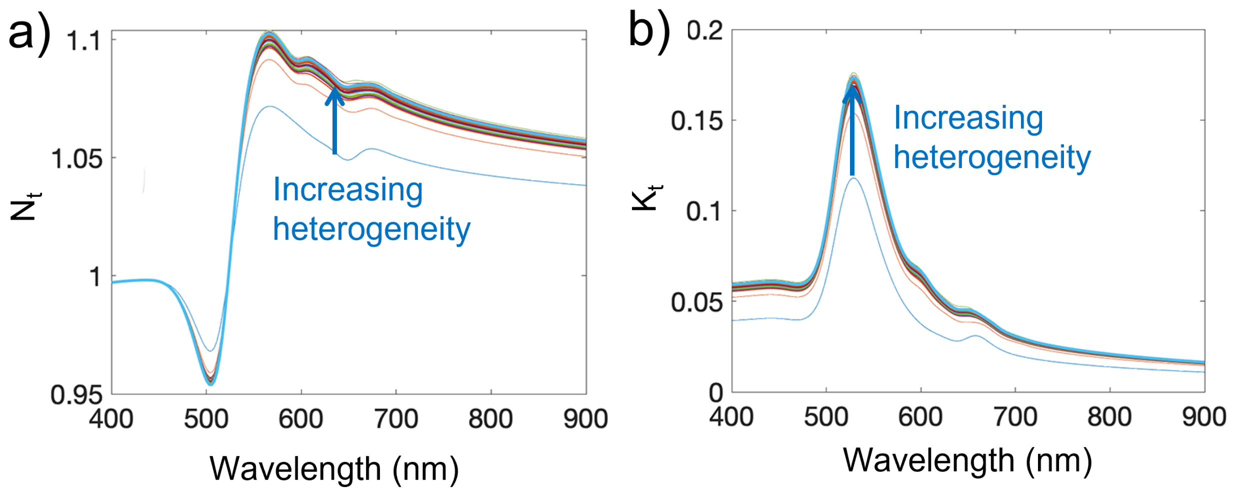


Figure S5: The real (a) and the imaginary (b) refractive index components, calculated for transverse polarization of the incident light, for composites with varying degrees of size heterogeneity as detailed in the text. The blue arrows show the direction of increased heterogeneity, where a broader range of size distributions is included in the calculations.

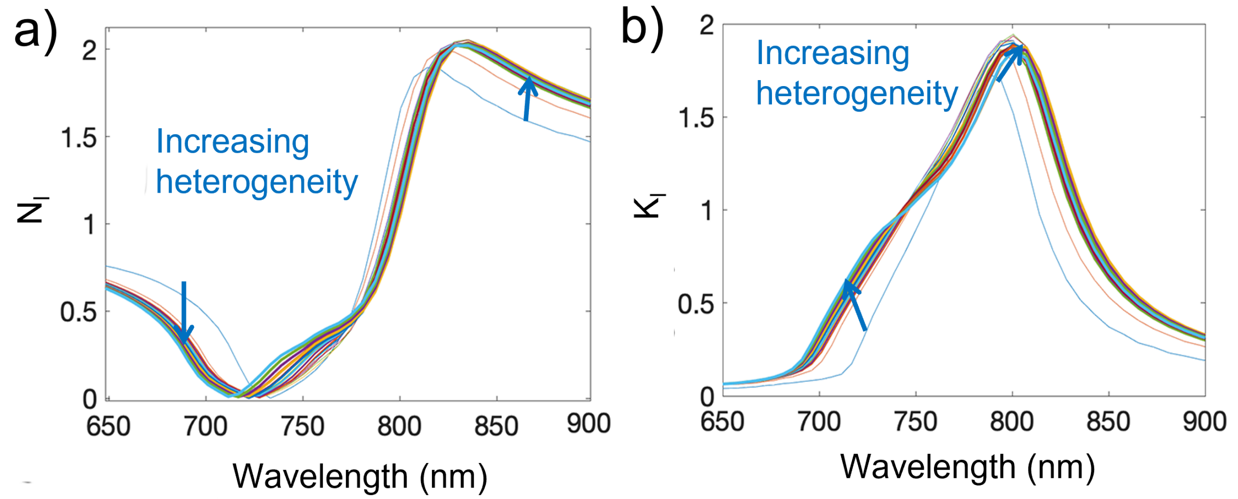


Figure S6: The real (a) and the imaginary (b) refractive index components, calculated for longitudinal polarization of the incident light, for composites with varying degrees of size heterogeneity as detailed in the text. The blue arrows show the direction of increased heterogeneity, were a broader range of size distributions is included in the calculations.

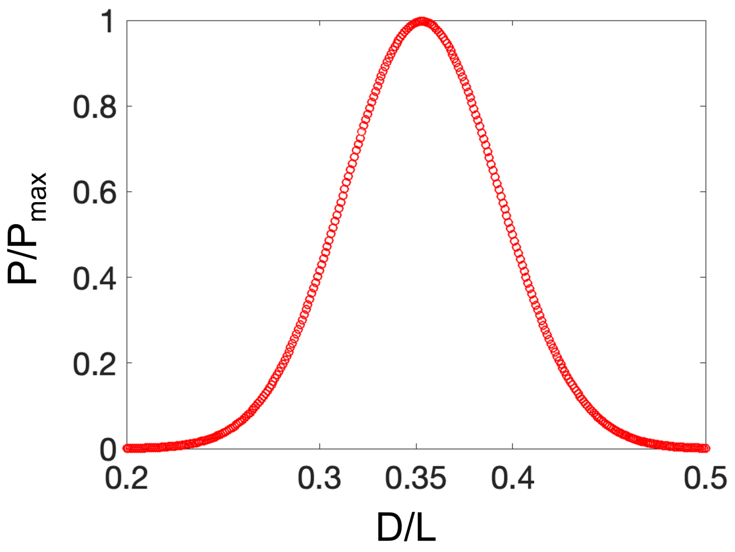


Figure S7: Gaussian distribution function ($N(\frac{12}{34}, 0.04^2)$) that models the sampling probability ($P$) of including AuNRs with varying aspect ratios in a heterogeneous sample. This distribution is centred around, $\frac{D}{L} = \frac{12}{34} = 0.35$, the average-sized AuNR.

As seen for the dipole moments in Figure S4, the real refractive indexes shown in Figure S5a and S6a, and the imaginary index components shown in Figure S5b and S6b, as the number of incorporated AuNR sizes increases, increasing heterogeneity, the calculated spectra red-shift and a right shoulder raises, meaning that the LSPR resonance broadens as size heterogeneity is increased.

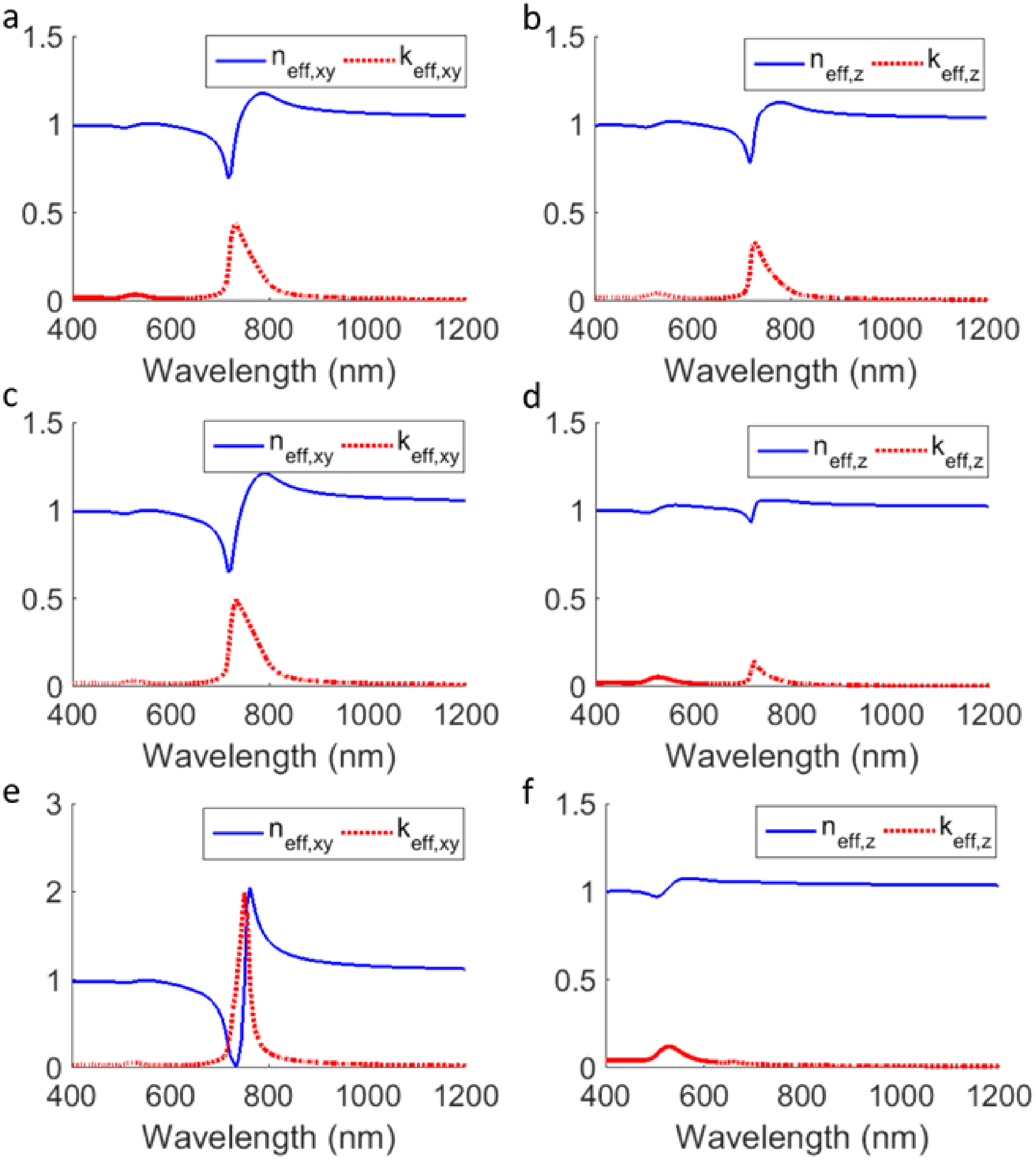


Figure S8: Effective index calculation for an AuNR/PMMA composites with predominantly in-plane rod orientation, assuming a flat distribution of angles between $\theta_{min} < \theta < 90°$, with (a,b) $\theta_{min} = 30°$, (c,d) $\theta_{min} = 60°$, and (e,f) $\theta_{min} = 90°$, respectively. The calculated orientation order parameter for these tilt angles, based on equation 8 of the main text is $S_z = -0.08 \pm 0.05$ for $\theta_{min} = 30°$, $S_z = -0.32$ for $\theta_{min} = 60°$, and $S_z = -0.5$ for $\theta_{min} = 90°$, respectively.

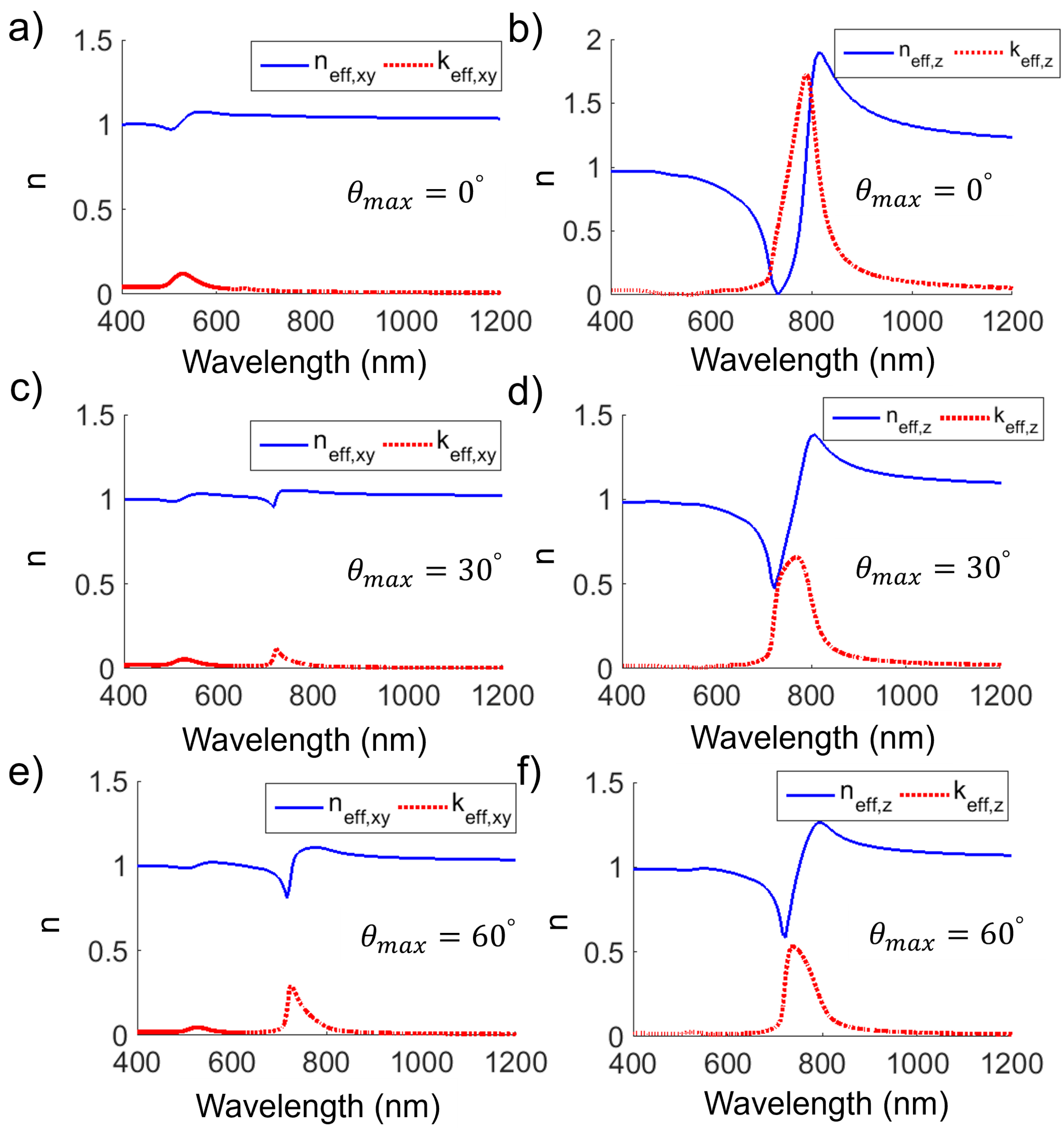


Figure S9: Effective index calculation for an AuNR/PMMA composite with predominantly out-of-plane rod orientation, assuming a flat distribution of angles between $0° < \theta < \theta_{max}$, with (a,b) $\theta_{max} = 0_{max}$, (c,d) $\theta_{max} = 30°$, and (e,f) $\theta_{max} = 60°$, respectively. The calculated orientation order parameter for these tilt angles, based on equation 8 of the main text is $S_z = 1$ for $\theta_{max} = 0°$, $S_z = 0.61$ for $\theta_{max} = 30°$, and $S_z = 0.25$ for $\theta_{max} = 60°$, respectively.

# Experimental Details

**AuNR synthesis.** AuNRs were synthesized using an aqueous seed-mediated growth method following previously reported procedures.[5–8] Briefly, for each batch 40 mL of DI water in a centrifuge tube was placed in the freezer. Additional centrifuge tubes were filled with 40 mL water, and placed in a 35° water bath. A second water bath was heated to 32° in preparation for the gold seed solution. Next, 21.1 mL of DI water was added to 83.1 mg of gold chloride ($AuCl_3$) in a clean, foil-wrapped vial to minimize its exposure. The solution was set aside for later use. In another centrifuge tube, 364 mg of Cetyltrimethyl ammonium bromide (CTAB) and 10 mL water were combined. The alternation between vortexing and sonicating was done until the solution was clear to ensure that the CTAB was completely dissolved. Then, 15.2 mg of $NaBH_4$ was measured into a scintillation vial and combined with the ice-cold Di water by rinsing the vial into the centrifuge tube to avoid static loss. This was done due to the exothermic nature of the reaction. The gold seed was made by combining the following in another clean, 7.5 mL CTAB solution in $H_2O$, 250 $\mu$L of $AuCl_3$ solution, and 600 $\mu$L of the $NaBH_4$ in ice cold DI water. After gently swirling, the solution was placed in the prepared 32° C water bath for 2 hours.

1.456 g CTAB was separately added to the centrifuge tubes held in the 35° C water bath. The bath temperature was turned down to 32° C, then the tubes were alternated between vortexing and sonication to ensure complete dissolution of the CTAB. Once the CTAB was well dispersed, the tubes were placed back in their 32° C water bath. Next, two more clean scintillation vials were wrapped in foil. In one, 5 mL of DI water was added to 88 mg of ascorbic acid. In the other foil-wrapped vial, 20 mL of DI water was added to 34 mg of $AgNO_3$, to minimize exposure to light. At the end of 2 hours, the following was micropipetted in order into the CTAB solutions waiting in the 32° C water bath: 1.7 mL of $AuCl_3$ solution in DI water, 250 $\mu$L of $AgNO_3$, 270 $\mu$L of the ascorbic acid solution, and 420 $\mu$L of the gold seed solution. The ascorbic acid and the gold seed were injected hard. Each tube was swirled a few times until the solution became colorless,

and then immersed in the 32° C water bath overnight. When the synthesis was successful, the solution would turn a dark reddish-purple hue. UV-Vis spectrometry was performed on a Cary 5000 UV-vis spectrometer on each batch to ensure a high reaction yield after 24 hours. The tubes were then centrifuged at 8500 rpm for 20 minutes in holders with holes at the bottom. The supernatant was removed, leaving about 10 mL in each batch. AuNRs were then stored in the CTAB solution at room temperature. Prior to use, the solutions were warmed to 32° C to redissolve the CTAB; UV-Vis spectra were taken again to assess the quality of the solution post storage.The size distribution of the AuNRs was estimated from the position of the LSPR band from UV-Vis spectroscopy and also directly determined from SEM measurements.[5,9] For SEM measurements, AuNRs were solution cast onto silicon substrates, and representative images were analyzed using ImageJ software. Roughly 1000 individual AuNRs were measured to accurately measure the distribution.

Thiolated PS brushes were grafted to the surface of the AuNRs as described elsewhere.[9] Briefly, 30 mg of the desired molecular weight thiol-terminated polystyrene (PS) was added to 5 mL of tetrahydrofuran (THF) in a scintillation vial and left to stir until the polymer was completely dissolved. Next, a 3mL aliquot of the AuNR solution was centrifuged for 30 min at 8500 rpm. The supernatant was then removed and the pellet was injected hard into the polymer/THF solution while stirring and then left overnight under heavy mixing. The THF destabilizes the native cetyltrimethylammonium bromide (CTAB) layer and facilitates the grafting of the thiolated brushes. For the end-to-end linked rods (Figure S13), four batches of AuNRs were mixed and centrifugated down to 100 $\mu$L as before. This 4× concentrated solution was then injected into a thiol-PS / THF mixture. The weakening of the native CTAB layer through the exchange to THF combined with the increased concentration of AuNRs facilitated end-linking as thiolated polymers are thought to have preferentially grafted to the barrel of the AuNRs. In all cases, the thiolated polymers were allowed to incubate with the AuNRs in THF overnight followed by three washings through centrifugation at 8500 rpm for 40 min, removal of supernatant, and finally a solvent exchange to 3 mL of toluene. A

post-grafting UV-vis was taken to ensure AuNRs remained well dispersed.

To make PNC solutions, grafted AuNRs were centrifuged again (8500 rpm, 30 min) and the pellet was injected into polymer/toluene solutions which were prepared by adding 0.7-0.4 mg of free polymer to 100 $\mu$L of AuNR solution in toluene. The final solution was filtered (0.45 $\mu$m) to remove any macroscopic impurities and then left overnight to settle and dissolve. 200 $\mu$L of the polymer/AuNR solution was then spun-cast at 2000 rpm onto cleaned Si wafer, clear glass, and frosted glass substrates for SEM, UV-vis, and VASE measurements, respectively. The samples were allowed to dry overnight prior to any imaging or spectroscopy. For the ultra-thin films (low wt% polymer), the substrates were UV-plasma cleaned prior to use to avoid dewetting. The measured film thicknesses were slightly larger than the AuNR diameter in all cases.

# Spectroscopic Ellipsometry Experiments

The details of SE fitting to multiple oscillator models were kept similar to our previous reports.[4] Briefly, the PNC layer was parameterized using a B-spline model that fits the data point by point with a resolution of 0.5 eV, in a Kramers-Kronig consistent format. The data was then fit to a multi-oscillator model (Gaussian or Lorentzian depending on the peak broadening) to account for the longitudinal and transverse LSPR resonances. Typically 2-3 oscillators were required, one for the longitudinal LSPR (1.4-1.6 eV), one for the transverse LSPR ( 2.3 eV), and one that was representative of rod coupling (1.3-1.4 eV). When the spectra were too broad due to size heterogeneity, additional overlapping oscillators were used to fully capture the calculated indices of refraction from the B-spline model. Once the layer was replaced, the data was fit again to this model to ensure that every feature was accounted for. Figure S10 shows an example of the raw ellipsometry data and isotropic fitting results. The complex index of refraction ($n = N + iK$) was calculated based on the complex dielectric constant ($\epsilon = \epsilon' + \epsilon$") as $n = \sqrt{\mu\epsilon}$ where $\mu = 1$ is the magnetic permeability of a non-magnetic

medium. The imaginary part of the index of refraction ($K$) is also the extinction coefficient of the material. Figure 2 of the main text shows that the calculated extinction coefficient ($K$) from ellipsometry fitting agrees well with the measured extinction coefficient using UV-vis spectroscopy (Figure 2c).

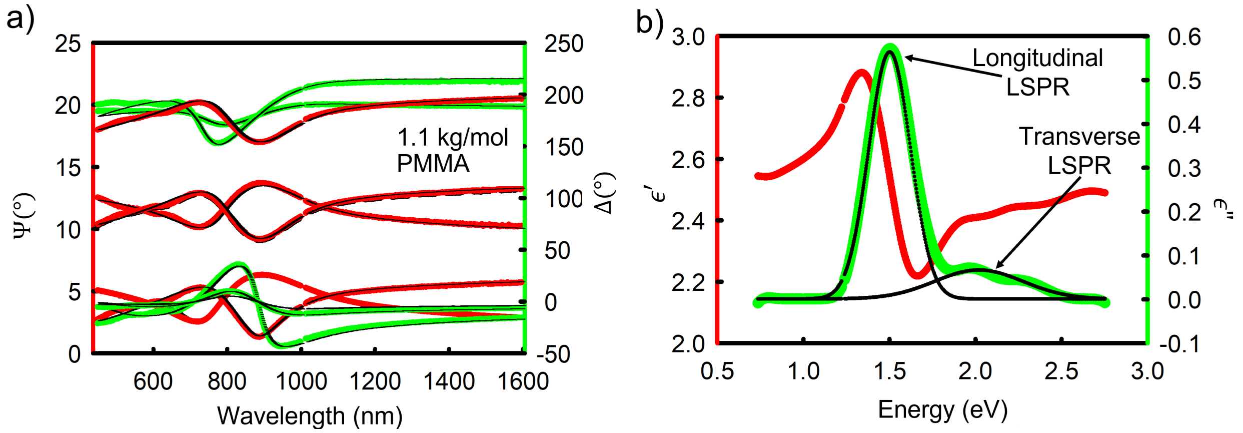


Figure S10: (a) Raw VASE data measuring SE angles Ψ (red, left axis) and Δ (green, right axis) at five different angles between 55° - 70° as a function of wavelength (nm) along with the fitted isotropic model (dashed line) globally fit to all data sets at all angles. Data was gathered on a thick, isotropic film made of well-dispersed AuNRs with 11.5 kg/mol PS brush in a 1.1 kg/mol PMMA matrix. (b) The calculated real ($\epsilon'$) and imaginary ($\epsilon$") parts of the dielectric constant. The black lines show the fitted oscillators for the longitudinal and transverse LSPR resonances as indicated by the black arrows.

The data was then fit to a birefringent model only allowing to fit for the amplitude of each resonance, keeping the resonance location and breadth constant. Before converting the fit to an anisotropic layer, one more adjustment was made. If a coupling oscillator was used, it was not used for fitting in the out-of-plane ($z$) direction, to avoid over-fitting. The fitting of the transverse LSPR to birefringence typically did not improve the fitting and as such was mostly kept constant with the same value in both directions. Figure S11 shows the calculated indices of refraction of anisotropic models on two example PNCs, with varying degrees of birefringence. The orientation order parameter $S_z$ was then calculated based on the value of the extinction coefficient $K$, at the longitudinal LSPR resonance, as described in the main text (Equation 8 of the main text) and schematically shown in Figure 2 of the main text and Figure S11.

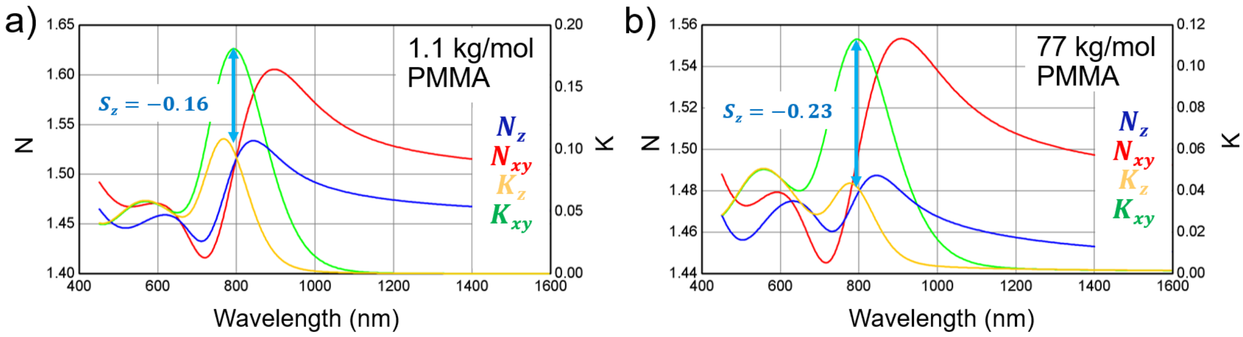


Figure S11: VASE measurements of the complex, birefringent indices of refraction. a) the in-plane real ($N_{xy}$, red) and imaginary ($K_{xy}$, green) and out-of-plane real ($N_z$, blue) and imaginary ($K_z$, yellow) indices of refraction vs. wavelength calculated based on variable-angle spectroscopic ellipsometry for a PNC composed of AuNRs with 20 kg/mol PS brush in a 1.1 kg/mol PMMA matrix. Based on these measurements, the film thickness is $h = 79 \pm 1$ nm and the LSPR peak is at 794 nm, matching the UV-vis data. The orientation order parameter is evaluated based on the extinction coefficients $K_{xy}$ and $K_z$ to be $S_z = -0.16 \pm 0.5$. b) Similar data was obtained on a film containing the same AuNRs in a 77 kg/mol PS matrix with a measured film thickness of $h = 54 \pm 1$ nm. The AuNRs are on average slightly more in-plane in this system resulting in $S_z = -0.23 \pm 0.5$.

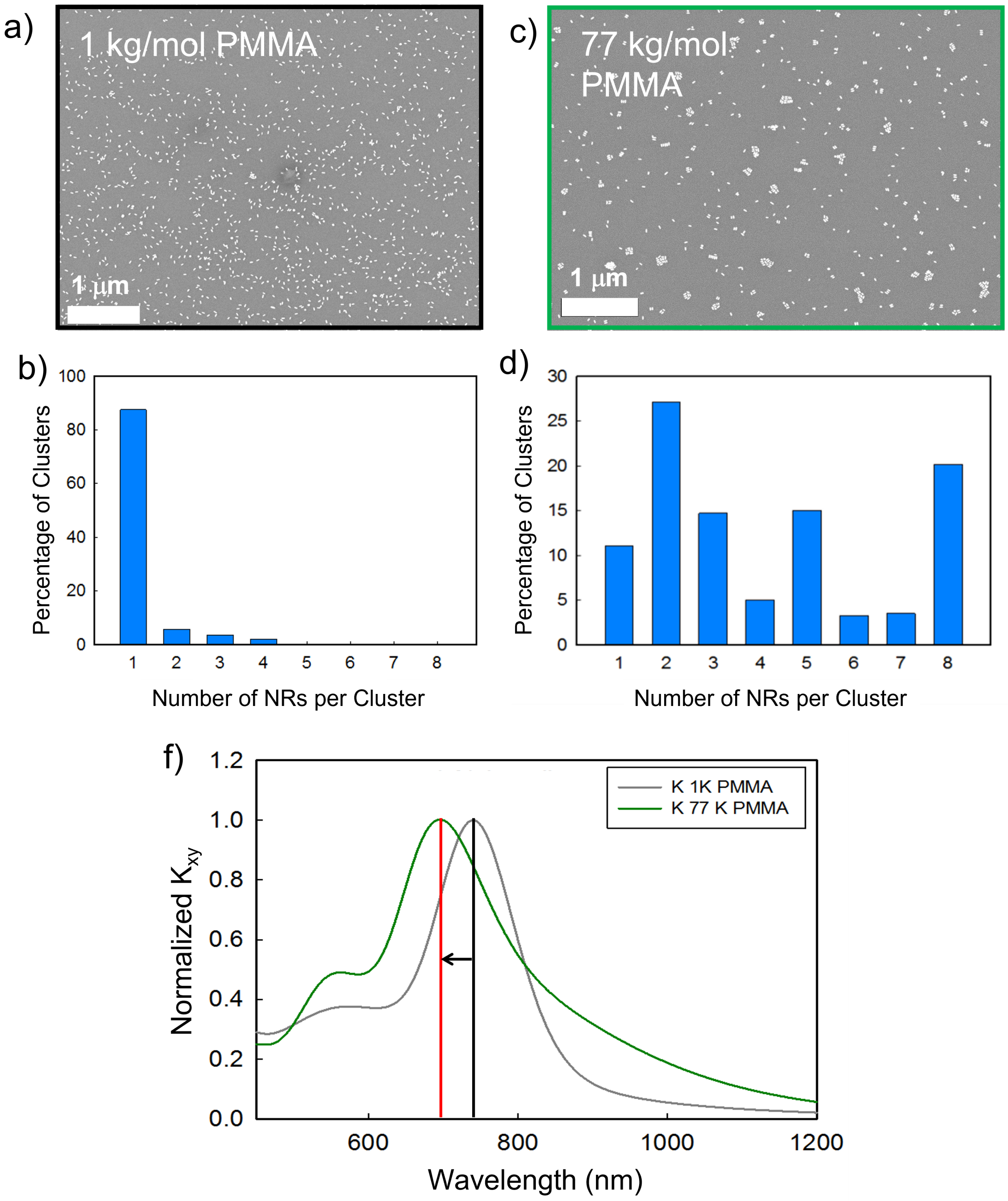


Figure S12: (a) SEM image of a PNC thin film with 4% loading of AuNRs with 11.5 kg/mol PS brushes in a 1 kg/mol PMMA matrix showing good dispersion along with (b) aggregation statistics. (c) SEM image of a PNC thin film with 4% loading of AuNRs with 11.5 kg/mol PS brushes in a 77 kg/mol PMMA matrix showing aggregation along with (d) aggregation statistics. (e) normalized extinction coefficients ($K_{xy}$) of the two samples measured using VASE showing a blue-shift in the LSPR peak commensurate with aggregation seen in the SEM images.

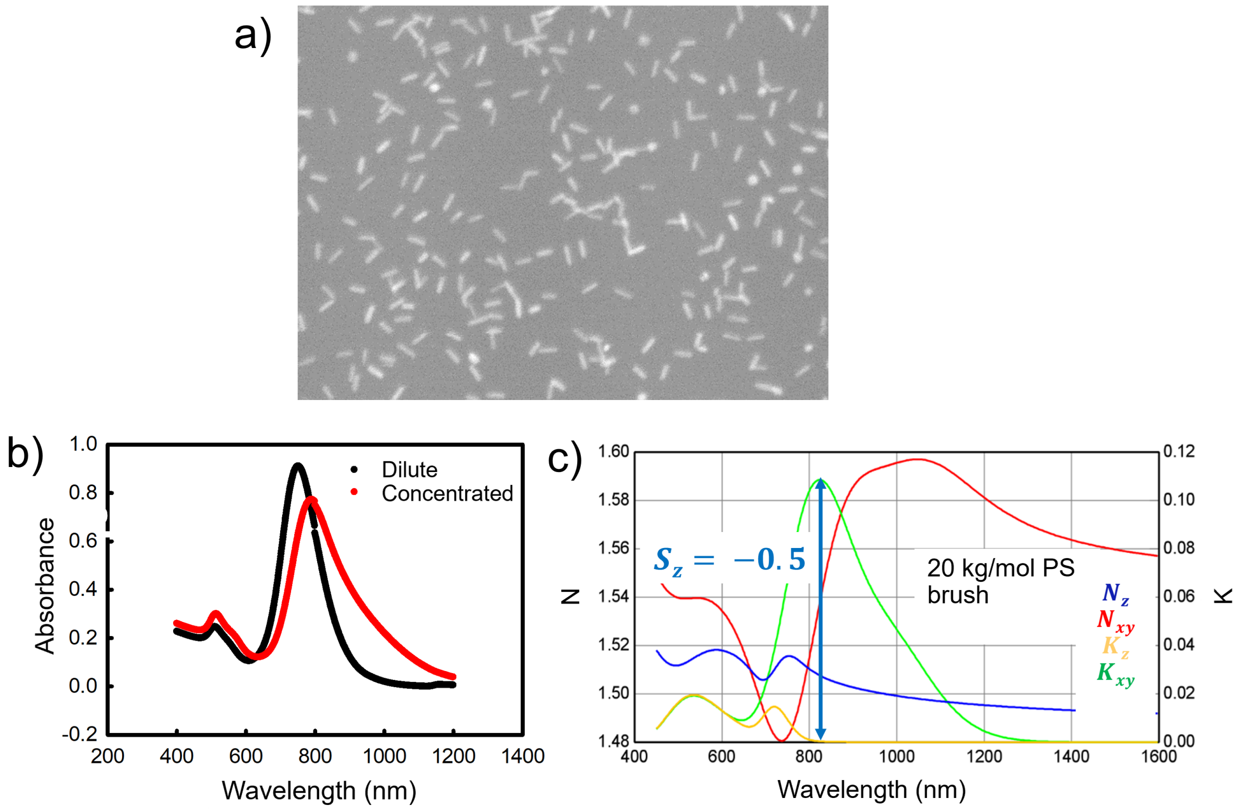


Figure S13: An example of perfectly in-plane AuNRs, produced by depositing a colloidal solution of rods on a silicon substrate. The solution was prepared at a high concentration of AuNRs, with 20 kg/mol PS brushes, resulting in end-to-end attachments of rods as seen in (a). (b) UV-vis spectra of dilute (black) and concentrated (red) solutions of AuNRs showing the red-shifting of the extinction coefficient due to end-to-end AuNR attachment at high concentration. (c) the in-plane ($N_{xy}$, $K_{xy}$) and out-of-plane ($N_z$, $K_z$) indices of refraction of the concentrated sample measured using VASE. The out-of-plane extinction ($K_z$) has no component representing the longitudinal LSRP peak, resulting in $S_z = 0$. The in-plane extinction ($K_{xy}$) shows a significant degree of red-shifting and double peaks in the longitudinal LSPR region, as well as an additional peak in the transverse region (wavelength less than 800 nm), indicating the end-to-end linking of AuNRs and significant heterogeneity of angles between linked in-plane AuNRs in the sample.

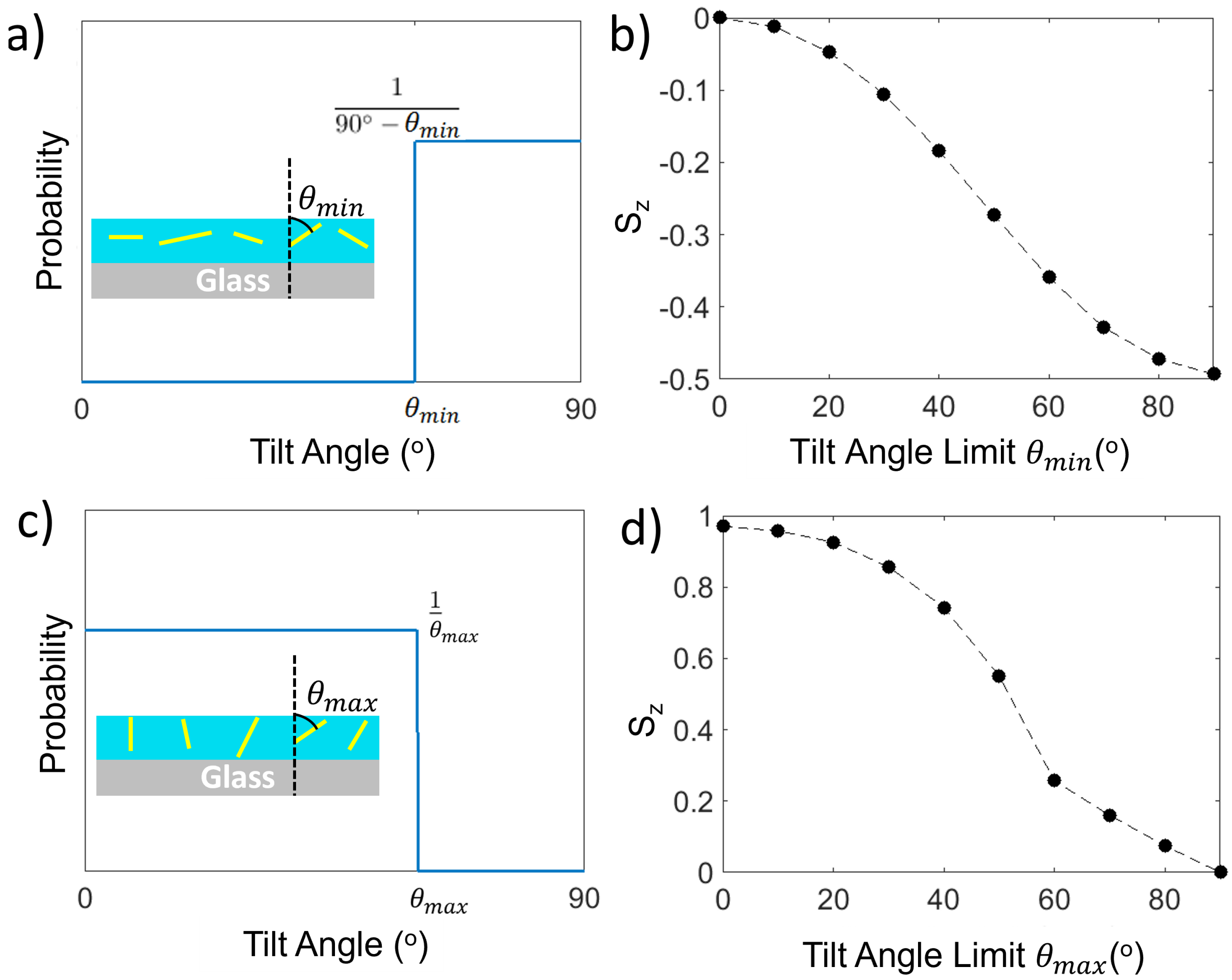


Figure S14: (a) Tilt angle distribution for mostly in-plane AuNRs, showing the definition of $\theta_{min}$. (b) The dependence of the orientation order parameter ($S_z$) on the value of $\theta_{min}$. (c) Tilt angle distribution for mostly out-of-plane AuNRs, showing the definition of $\theta_{max}$. (b) The dependence of the orientation order parameter ($S_z$) on the value of $\theta_{max}$.

# References


(1) Papas, C. H. *Theory of electromagnetic wave propagation*; Dover: New York, 2011.

(2) Shafiei, F.; Monticone, F.; Le, K. Q.; Liu, X.-X.; Hartsfield, T.; Alù, A.; Li, X. A sub-wavelength plasmonic metamolecule exhibiting magnetic-based optical Fano resonance. *Nature nanotechnology* **2013**, *8*, 95–99.

(3) Baysinger, G.; Berger, L. I.; Goldberg, R.; Kehiaian, H.; Kuchitsu, K.; Rosenblatt, G.;

Roth, D.; Zwillinger, D. CRC handbook of chemistry and physics. *National Institute of Standards and Technology* **2015**,

(4) Glor, E. C.; Ferrier, R. C.; Li, C.; Composto, R. J.; Fakhraai, Z.; Chen, S. H.; Troester, M.; Tracy, J.; Oldenburg, A.; Klinge, L. et al. Out-of-plane orientation alignment and reorientation dynamics of gold nanorods in polymer nanocomposite films. *Soft Matter* **2017**, *54*, 301–307.

(5) Ferrier Jr, R. C.; Lee, H.-S.; Hore, M. J.; Caporizzo, M.; Eckmann, D. M.; Composto, R. J. Gold nanorod linking to control plasmonic properties in solution and polymer nanocomposites. *Langmuir* **2014**, *30*, 1906–1914.

(6) Nikoobakht, B.; El-Sayed, M. A. Preparation and growth mechanism of gold nanorods (NRs) using seed-mediated growth method. *Chemistry of Materials* **2003**, *15*, 1957–1962.

(7) Sau, T. K.; Murphy, C. J. Seeded high yield synthesis of short Au nanorods in aqueous solution. *Langmuir* **2004**, *20*, 6414–6420.

(8) Hore, M. J.; Composto, R. J. Nanorod self-assembly for tuning optical absorption. *ACS nano* **2010**, *4*, 6941–6949.

(9) Ferrier Jr, R. C.; Koski, J.; Riggleman, R. A.; Composto, R. J. Engineering the assembly of gold nanorods in polymer matrices. *Macromolecules* **2016**, *49*, 1002–1015.
